# White matter changes in chronic and episodic migraine: a diffusion tensor imaging study

**DOI:** 10.1186/s10194-019-1071-3

**Published:** 2020-01-02

**Authors:** Álvaro Planchuelo-Gómez, David García-Azorín, Ángel L. Guerrero, Santiago Aja-Fernández, Margarita Rodríguez, Rodrigo de Luis-García

**Affiliations:** 1 Imaging Processing Laboratory, Universidad de Valladolid, Valladolid, Spain; 2 Headache Unit, Department of Neurology, Hospital Clínico Universitario de Valladolid, Avenida Ramón y Cajal 3, 47005 Valladolid, Spain; 3 Institute for Biomedical Research of Salamanca (IBSAL), Salamanca, Spain; 4 Department of Medicine, Universidad de Valladolid, Valladolid, Spain; 5 Department of Radiology, Hospital Clínico Universitario de Valladolid, Valladolid, Spain

**Keywords:** Migraine, Chronic migraine, Diffusion tensor imaging, Magnetic resonance imaging (MRI), Tract-based spatial statistics

## Abstract

**Background:**

White matter alterations have been observed in patients with migraine. However, no microstructural white matter alterations have been found particularly in episodic or chronic migraine patients, and there is limited research focused on the comparison between these two groups of migraine patients.

**Methods:**

Fifty-one healthy controls, 55 episodic migraine patients and 57 chronic migraine patients were recruited and underwent brain T1-weighted and diffusion-weighted MRI acquisition. Using Tract-Based Spatial Statistics (TBSS), fractional anisotropy, mean diffusivity, radial diffusivity and axial diffusivity were compared between the different groups. On the one hand, all migraine patients were compared against healthy controls. On the other hand, patients from each migraine group were compared between them and also against healthy controls. Correlation analysis between clinical features (duration of migraine in years, time from onset of chronic migraine in months, where applicable, and headache and migraine frequency, where applicable) and Diffusion Tensor Imaging measures was performed.

**Results:**

Fifty healthy controls, 54 episodic migraine and 56 chronic migraine patients were finally included in the analysis. Significant decreased axial diffusivity (*p* < .05 false discovery rate and by number of contrasts corrected) was found in chronic migraine compared to episodic migraine in 38 white matter regions from the Johns Hopkins University ICBM-DTI-81 White-Matter Atlas. Significant positive correlation was found between time from onset of chronic migraine and mean fractional anisotropy in the bilateral external capsule, and negative correlation between time from onset of chronic migraine and mean radial diffusivity in the bilateral external capsule.

**Conclusions:**

These findings suggest global white matter structural differences between episodic migraine and chronic migraine. Patients with chronic migraine could present axonal integrity impairment in the first months of chronic migraine with respect to episodic migraine patients. White matter changes after the onset of chronic migraine might reflect a set of maladaptive plastic changes.

## Background

According to the III^rd^ edition of the International Classification of Headache Disorders (ICHD-3), patients with Chronic Migraine (CM) suffer from headache during 15 or more days per month for more than 3 months, with at least eight of these days with migrainous characteristics [1]. Between 2 and 3% of migraine patients evolve annually from Episodic Migraine (EM) to CM [2]. Some risk factors have been associated with progression from EM to CM, but the pathophysiological mechanisms of this conversion remain to be elucidated. Moreover, EM and CM could represent either two ranges of the same entity, or two subgroups with distinctive characteristics. Previous review studies have exposed that important distinctions exist along the continuum between EM and CM [3, 4], but it is not clear whether CM is a kind of more frequent EM or a distinct entity.

Magnetic Resonance Imaging (MRI) is one of the most powerful technologies available for the study of the migrainous brain. Among its different modalities, diffusion MRI (dMRI) is particularly well suited for the analysis of possible white matter alterations in migraine. dMRI studies have shown changes affecting the white matter in migraineurs with respect to healthy controls; these changes were mainly observed in the corpus callosum [5–11], thalamus [7, 8, 12, 13], thalamic radiation [7–9, 14, 15] and cingulate gyrus [9, 10, 15–17].

Some studies using dMRI have focused on migraine with and without aura. Even though significant differences have been found [10, 18], using MRI information it remains unclear whether migraine with and without aura area are actually two distinct entities, the manifestations of the same pathophysiological substrate on two different phenotypes [19, 20], or even whether aura can be better defined as a migraine phase, but not a category such as chronic and episodic migraine [21].

Some other studies have employed dMRI to investigate CM. To the best of our knowledge, however, only one study by Neeb et al. searched for possible differences between patients with EM and CM, not finding any significant differences [22]. Other studies compared chronic migraine patients with healthy controls [16] or mixed EM and CM patients in order to compare migraine patients and controls [14]. Previous studies alternatively used T1-weighted MRI images [23, 24] and functional MRI [25], respectively, to compare possible differences between patients with CM and EM. These studies described reduced grey matter volume in CM compared to EM [23] and more activity in CM compared to EM patients with headaches during the scanning in the anterior hypothalamus [25].

The present study performs a detailed comparison of the white matter in EM, CM and healthy controls over a large cohort of subjects, using dMRI data. We hypothesised that there could be white matter structural differences between CM and EM patients, although no significant differences were found in a previous study [22]. In our study, we included a considerably higher number of patients and controls compared to that study. Our goals are to:

1. Investigate whether there are significant differences between CM and EM, and between these groups and healthy controls. To that end, TBSS [26] was employed as dMRI analysis technique.
2. Examine how white matter descriptors based on dMRI relate to clinical features in migraine patients. To that end, a correlation analysis between dMRI parameters and variables such as duration of migraine and time from onset of CM was performed.

## Materials and methods

### Participants

We conducted an observational analytic study with a case-control design. The target population included patients with migraine. Patients were firstly screened and recruited from the headache outpatient unit at the Hospital Clínico Universitario de Valladolid (Valladolid, Spain), a tertiary centre that receives patients both from specialized care and directly from primary care. Inclusion criteria were: a) Diagnosis of episodic or chronic migraine according to the ICHD-3 beta and ICHD-3 criteria [1, 27]; b) no changes in the situation of episodic or chronic migraine in the previous 3 months; c) agreeing to participate and signing the Informed Consent; d) aged between 18 and 60. We excluded patients with a) high frequency Episodic Migraine, suffering between 10 and 14 headache days per month (to avoid any confusion between high frequency EM and CM [28]); b) other non-craniofacial painful conditions occurring 10 or more days per month; c) known major psychiatric diseases (described in anamnesis or presence of Depression or Anxiety according to Hospital Anxiety and Depression Scale [29]); d) other neurological diseases; e) drug or substance abuse; f) pregnancy or childbearing; g) other headache disorders. All the patients scanned were preventive naïve. Patients were asked to keep a headache diary for 3 months (before inclusion) and were classified as EM when they had less than 10 headaches per month, or CM according to ICHD-3 criteria. Participants with EM were not allowed to have headache days with tension-type headache (TTH) phenotype. No healthy controls (HC) were included if they showed a present or past history of migraine, or if any other neurological or psychiatric condition was present, with the sole exception of infrequent TTH. We used a non-probabilistic sampling method by convenience sampling. Healthy controls balanced for age and sex were recruited through hospital and University colleagues and advertisements in these facilities by convenience sampling and snowball sampling.

For all patients, sociodemographic and clinical data were collected, including the duration of migraine disease (years), headache and migraine frequency (days per month) and time from the onset of chronic migraine (months) when applicable. The intake of symptomatic medication, i.e., combination of analgesics and triptan, was considered to verify if patients fulfilled criteria of acute medication overuse (intake at 10 or more days per month). Presence of aura was drawn.

The local Ethics Committee of Hospital Clínico Universitario de Valladolid approved the study (PI: 14–197). All participants read and signed a written consent form prior to their participation.

### MRI acquisition

Images were acquired for migraine patients after at least 24 h from the last migraine attack. High-resolution 3D T1-weighted and diffusion-weighted MRI data were acquired using a Philips Achieva 3 T MRI unit (Philips Healthcare, Best, The Netherlands) with a 32-channel head coil in the MRI facility at the Universidad de Valladolid (Valladolid, Spain).

For the anatomical T1-weighted images, the following acquisition parameters were used: Turbo Field Echo (TFE) sequence, repetition time (TR) = 8.1 ms, echo time (TE) = 3.7 ms, flip angle = 8°, 256 × 256 matrix size, 1 × 1 × 1 mm^3^ of spatial resolution and 160 slices covering the whole brain.

Diffusion-weighted images (DWI) were acquired using the next parameters: TR = 9000 ms, TE = 86 ms, flip angle = 90°, 61 gradient directions, one baseline volume, b-value = 1000 s/mm^2^, 128 × 128 matrix size, 2 × 2 × 2 mm^3^ of spatial resolution and 66 axial slices covering the whole brain.

T1 and diffusion-weighted scans were acquired during the same session, starting with the T1 scan followed by the diffusion-weighted scan, between May 2014 and July 2018. Total acquisition time for each subject was around 18 min.

### Image processing

MR images were processed before carrying out the statistical analysis using TBSS [26]. For the TBSS analysis, four Diffusion Tensor Imaging (DTI) measures were obtained: Fractional Anisotropy (FA), Mean Diffusivity (MD), Radial Diffusivity (RD) and Axial Diffusivity (AD). In a nutshell, FA reflects the degree of directionality of water diffusivity, MD is a global measure of water diffusion, RD quantifies the diffusion perpendicular to the principal direction and AD is the diffusion in the main direction of the white matter fibres [30].

Prior to the obtention of the four DTI measures, diverse preprocessing procedures were implemented on the DWI data. Diffusion-weighted images were denoised, using “dwidenoise” tool from MRtrix [31, 32], eddy currents and motion corrected, using “dwipreproc” tool from MRtrix [33], and B1 field inhomogeneity corrected, using “dwibiascorrect” tool with the “-fast” option from MRtrix [34, 35].

Once the DWI images were preprocessed, a whole brain mask for each image was generated using “dwi2mask” tool from MRtrix [36] and, next, diffusion tensors at each voxel were estimated using the “dtifit” tool from FSL [37], also obtaining FA, MD and AD maps. RD was manually calculated by obtaining the mean of the second and the third eigenvalues, which were also previously computed with “dtifit”.

For the TBSS method, all participants’ FA images were nonlinearly registered using the FNIRT tool from FSL to a template of the averaged FA images (FMRIB-58) in Montreal Neurological Institute (MNI) space; the FNIRT tool uses a b-spline representation of the registration warp field [38]. After registration, a mean FA image was generated and thinned to create a mean FA skeleton of white matter tracts using a FA value of .2 as threshold to distinguish white from grey matter. Then, each subject’s aligned FA images were projected onto the mean FA skeleton. In a similar way, the TBSS process was repeated for MD, AD and RD, using the protocol devoted for non-FA images. To identify the white matter tracts, the Johns Hopkins University ICBM-DTI-81 White-Matter Labels Atlas [39, 40] provided in the FSL toolbox was used. However, this atlas does not cover the whole white matter across brain, so we also employed the Johns Hopkins University White-Matter Tractography Atlas [41], which contains a lower number of tracts but covers areas not included in the other atlas. The minimum volume to consider significant results in a region was set to 30 mm^3^. It must be noted that, because we use an image of 1 mm^3^ in the MNI space to identify the regions, the volume in mm^3^ is equal to the number of voxels. Moreover, when we extract the significant results in a region, we consider all the significant voxels from that region, from one or more clusters.

### Statistical analysis

We estimated Sample Size according to Chong and Schwedt, 2015 [14]. Based on the FA results from this study in three major tracts, we calculated a worst possible scenario model with an estimated effect size of difference between groups of .02 (greatest difference between groups) and a variance of .003 (greatest single group variance); a type 1-error rate of 1% and 80% power and anticipating a proportion of 10% of lost patients. The expected sample size was 167 participants.

Kolmogorov-Smirnov and Levene’s Test for equality of variances tests were used to assess normality and homogeneity of variance in age and duration of migraine in years. To test for significant differences in the age of the three groups, a one-way ANOVA was used if the null hypothesis in Kolmogorov-Smirnov and Levene tests was not rejected; otherwise, Kruskal-Wallis test was employed. To test for significant gender differences, a chi-square test was used. To compare continuous clinical features between migraine patients (i.e., duration of migraine history in years for both groups of patients and time from onset of chronic migraine in months for chronic migraine patients), a two-tailed unpaired t-test was used if the null hypothesis in Kolmogorov-Smirnov and Levene tests was not rejected; otherwise, Mann-Whitney U test was employed. To compare categorical clinical features between migraine patients, Fisher’s exact test was employed.

We executed group-wise comparisons of all migraineurs vs. healthy controls, CM vs. EM, EM vs. healthy controls, and CM vs. healthy controls. The voxel-wise TBSS differences in FA, MD, AD and RD values of white matter between the different groups were tested using a permutation-based inference tool by nonparametric statistics called “randomise”, implemented in FSL, with the threshold-free cluster enhancement (TFCE) option [42, 43]. Five thousand permutations were set to allow robust statistical inference and the significance threshold for intergroup differences was *p* < .05 after correcting for family wise error (FWE) applying the TFCE option. Additional clinical covariates were added to the comparisons in the cases where significant differences were found. These covariates were analysed individually to evaluate the individual effect of each covariate. In the case of presence of aura, we repeated the original TBSS analysis excluding the patients with migraine with aura. None of the design matrices included duration of migraine and time from onset of CM simultaneously as covariates due to collinearity. Time from onset of CM was also included as a covariate (only in comparisons with CM) because it may correct the results in CM patients in a more meaningful way than the total duration of migraine.

In the cases where significant differences were found, we performed a post-hoc analysis. We applied a false discovery rate (FDR) correction, using the “fdr” command from FSL, to the TFCE uncorrected *p*-values. The fdr command provides the uncorrected *p*-value which sets the level of statistical significance after the FDR correction. To correct for number of contrasts in each case, the final level of statistical significance is equal to the uncorrected p-value from the previous step divided by the number of comparisons (Bonferroni correction).

Effect size was computed using Cohen’s d value in regions with significant results from the first analysis (FWE-corrected). For every comparison, the mean value of the “most disabled” group was subtracted from the mean value of the “least disabled” or the control group. In the comparisons between both types of migraine, CM is considered the most disabled group, and EM the least disabled group.

To study the relationship between clinical parameters and DTI measures, Spearman’s rank correlation coefficient was employed in a ROI-based correlation analysis. Duration of the migraine in both types of migraine patients, time from onset of CM in chronic migraineurs, and headache and migraine frequency for both types of migraine patients were the analysed clinical parameters. It must be clarified that we obtained correlation values in CM and EM patients separately, in order to assess differences or trends within each type of migraine. Our intention was to determine the possible effect of headache or migraine frequency, in the specific range of episodic or chronic migraine, and the possible relationship with time, with special attention to the time from onset of CM. To obtain individual label maps for each subject, the inverse warp fields of the FA images to the MNI image transformation from the TBSS procedure were computed and applied to the Johns Hopkins University ICBM-DTI-81 White Matter Atlas. The ROIs that were selected for the correlation analysis were those for which significant differences were found between at least two groups in any diffusion parameter in the TBSS analysis. All DTI measures (FA, MD, RD and AD) were considered for the correlation analysis. We used all DTI measures to avoid a possible loss of complementary information given by each parameter in the assessment of differences within CM or EM. To correct for multiple comparisons, the Benjamini-Hochberg [44] FDR procedure was applied, and, after this correction, the level of statistical significance was set at *p* < .05.

## Results

During the study period, 51 healthy controls, 55 episodic migraine patients and 57 chronic migraine patients were recruited for the study after matching the inclusion and exclusion criteria. No significant structural abnormality was detected in conventional MRI studies. Due to erroneous results after applying the nonlinear registration to the FMRIB-58 image in MNI space, one healthy control, one episodic migraineur and one chronic migraineur were excluded from the study. Demographic and clinical data for the three groups with the remaining participants are summarised in Table 1. Significant differences were found in duration of migraine history in years between the two migraine groups and, as expected, in headache and migraine frequency between the migraine groups.

**Table 1:** Clinical and demographic characteristics of healthy controls (HC), episodic migraine (EM) and chronic migraine (CM)

	HC (*n* = 50)	EM (*n* = 54)	CM (*n* = 56)	Statistical test
Gender, male/female	11/39 (22/78%)	9/45 (17/83%)	6/50 (11/89%)	χ^2^_(2, *N* = 160)_ = 2.48, *p* = .29^†^
Age (years)	36.1 ± 13.2	37.1 ± 8.2	38.1 ± 8.7	χ^2^ (2) = 2.85, *p* = .24^‡^
Duration of migraine history (years)		14.1 ± 11.1	19.6 ± 10.4	t_(108)_ = − 2.7, *p* = .008^§^
Time from onset of chronic migraine (months)			24.5 ± 32.9	
Headache frequency (days/month)		3.6 ± 1.9	23.3 ± 6.3	U = 44.0, *p* < .001^¶^
Migraine frequency (days/month)		3.6 ± 1.9	13.9 ± 6.9	U = 108.5, *p* < .001^¶^
Overusing medication		0 (0%)	42 (75%)	*p* < .001^⁑^
Aura		9 (17%)	1 (2%)	*p* = .007^⁑^

^†^Chi-square test. ^‡^Kruskal-Wallis test. ^§^Two-tailed, unpaired Student’s t-test. ^¶^Mann-Whitney U test. ^⁑^Fisher’s exact test. Data are expressed as means ± SD

Considering the significant differences in duration of migraine history between episodic and chronic migraine patients (Table 1), TBSS analysis was repeated including the duration of migraine history as a covariate, as mentioned in the Statistical Analysis section. Presence of aura was added as an additional covariate to duration of migraine history in a posterior analysis. In the case of CM patients, the TBSS analysis was additionally accomplished including the time from onset of CM as a covariate. This covariate was included in a separate analysis from the one with duration of migraine history due to collinearity, as mentioned previously in the Statistical Analysis section.

### TBSS analysis

#### Results uncorrected for covariates

**Fig. 1:**
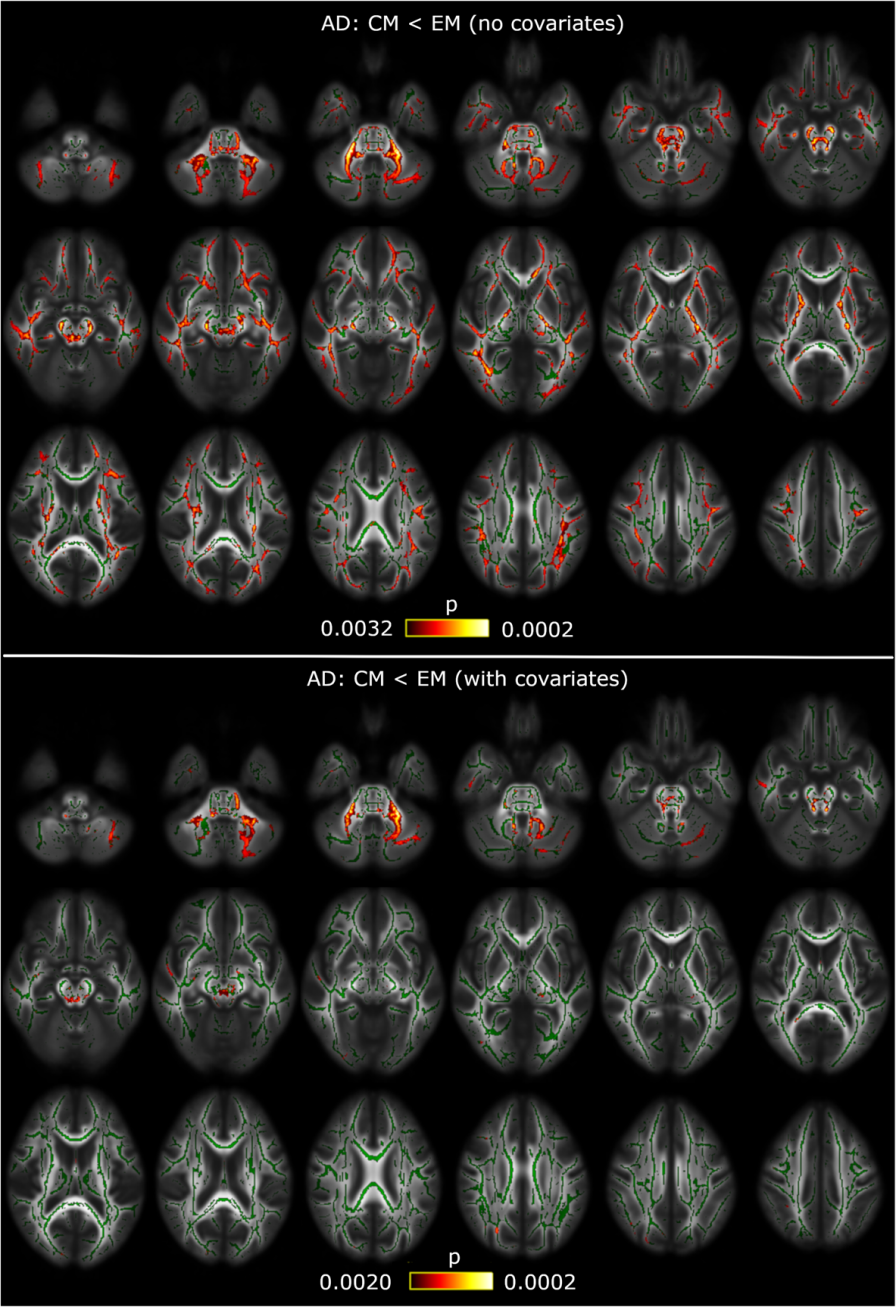
White matter alterations in chronic migraine compared to episodic migraine patients. TBSS shows decreased AD values in CM compared to EM in widespread locations with no covariate corrections (top) and correcting for time from onset of CM (bottom). White matter skeleton is shown in green, and voxels with significant differences in red-yellow. The colour bar shows the *p*-values (uncorrected). The maximum uncorrected p-value for each case is given by FDR and number of contrasts corrections

No significant differences were found in any of the diffusion indices (FA, MD, RD and AD) between all migraineurs and HC. Dividing all migraineurs into EM and CM, no significant differences were found with respect to HC. With regard to the comparison between CM and EM, no significant differences were found in FA, MD or RD. However, significant lower AD values were found in CM compared to EM in widespread locations across the white matter. These locations correspond to 38 different regions from the ICBM-DTI-81 White Matter Atlas, and six regions from the White Matter Tractography Atlas, and are shown in Tables 2 and 3 and Fig. 1. The FWE-corrected results can be seen in Additional file 1: Figure S1 and Table S1.

**Table 2:** White matter regions where significant decreased AD values were found in CM compared to EM

White Matter tract	Minimum *p*-value (uncorrected)	Volume (mm^3^)	MNI peak coordinate (mm), (x,y,z)
Middle cerebellar peduncle	.0002	2263	(−20,-55,-32)
Superior cerebellar peduncle R/L	.0002/.0002	145/137	(6,-31,-19) / (−6,-50,-27)
Inferior cerebellar peduncle R/L	.0002/.0002	81/118	(9,-42,-38) / (−13,-45,-31)
Superior longitudinal fasciculus R/L	.0004/.0004	565/821	(37,-48,14) / (−37,-50,15)
Genu of corpus callosum	.0006	154	(−8,27,1)
Body of corpus callosum	.0016	74	(−11,-19,30)
Splenium of corpus callosum	.0006	203	(21,-48,10)
Anterior corona radiata R/L	.0014/.0004	105/527	(19,24,-10) / (−18,25,-8)
Superior corona radiata R/L	.0012/.0014	253/87	(21,-9,34) / (−27,-10,25)
Posterior corona radiata R/L	.0010/.0010	75/140	(28,-40,21) / (−30,-52,22)
External capsule R/L	.0006/.0004	400/640	(33,-3,3) / (−22,16,-12)
Posterior limb of internal capsule R/L	.0010/.0008	372/400	(17,-4,8) / (−18,-1,10)
Retrolenticular part of internal capsule R/L	.0012/.0012	173/161	(30,-24,2) / (−24,-24,2)
Anterior limb of internal capsule R/L	.0006/.0004	163/270	(18,17,-3) / (−14,8,0)
Sagittal stratum R/L	.0002/.0006	418/316	(40,-36,-13) / (−40,-15,-14)
Posterior thalamic radiation R/L	.0004/.0002	333/254	(34,-56,3) / (−38,-52,3)
Cerebral peduncle R/L	.0004/.0002	225/257	(11,-22,-21) / (−10,-13,-12)
Corticospinal tract R/L	.0002/.0004	107/164	(10,-22,-23) / (−7,-19,-24)
Medial lemniscus R/L	.0002/.0002	83/103	(5,-35,-37) / (−2,-37,-30)
Pontine crossing tract	.0002	89	(7,-29,-25)
Fornix (cres) R/L	.0004/.0006	71/41	(33,-8,-17) / (−34,-11,-16)
Cingulum (hippocampus) L	.0002	87	(−17,-43,-2)

The column Volume represents the volume from the atlas region with significant differences (FDR-corrected and number of contrasts corrected). The maximum significant uncorrected p-value after corrections was .0032. No regions with volume equal or lower than 30 mm^3^ were included in this Table. Only regions with FWE-corrected *p* < .05 are included

*L* Left, *R* Right

**Table 3:** White matter regions where significant decreased AD values were found in CM compared to EM using the Johns Hopkins University White-Matter Tractography Atlas

White Matter tract	Minimum *p*-value (uncorrected)	Volume (mm^3^)	MNI peak coordinate (mm), (x,y,z)
Anterior thalamic radiation L/R	.0006/.0008	36/39	(−20,17,0) / (9,-30,-15)
Corticospinal tract L/R	.0012/.0012	152/165	(−21,-21,2) / (10,-24,-25)
Forceps major	.0010	126	(−17,-85,7)
Inferior longitudinal fasciculus R	.0004	49	(40,-35,-14)

The column Volume represents the volume from the atlas region with significant differences (FDR-corrected and number of contrasts corrected). The maximum significant uncorrected p-value after corrections was .0032. No regions with volume equal or lower than 30 mm^3^ were included in this Table

*L* Left, *R* Right

In the case of the analysis including only patients with migraine without aura, no new significant results were observed. As in the original sample, significant lower AD values were found in CM compared to EM (FWE-corrected results), but in 10 regions from the ICBM-DTI-81 White Matter Atlas, all of them included in the 38 regions with significant differences. The FWE-corrected results excluding migraine with aura patients can be seen in Additional file 1: Table S2.

#### Results corrected for covariates

After the post-hoc analysis, significant lower AD values were found in CM compared to EM in six regions from the ICBM-DTI-81 White Matter Atlas, and one region (the right corticospinal tract) from the White-Matter Tractography Atlas, when including the time from onset of CM as a covariate. These results are shown in Tables 4 and 5 and Fig. 1. No significant results were observed when correcting for total duration of migraine or for the other DTI measures.

**Table 4:** White matter regions from the ICBM-DTI-81 White Matter Atlas for which significant decreased AD values were found in CM compared to EM considering the effect of time from onset of CM

White Matter tract	Minimum *p*-value (uncorrected)	Volume (mm^3^)	MNI peak coordinate (mm), (x,y,z)
Middle cerebellar peduncle	.0004	1286	(−16,-52,-30)
Superior cerebellar peduncle R/L	.0004/.0002	97/109	(9,-50,-30) / (−6,-42,-26)
Inferior cerebellar peduncle L	.0006	55	(−6,-53,-24)
External capsule L	.0012	34	(−33,-10,1)
Pontine crossing tract	.0006	39	(0,-23,-24)

The column Volume represents the volume from the atlas region with significant differences (FDR-corrected and number of contrasts corrected). The maximum significant uncorrected p-value after corrections was .0020. No regions with volume equal or lower than 30 mm^3^ were included in this Table. Only regions with FWE-corrected *p* < .05 are included

*L* Left, *R* Right

**Table 5:** White matter regions from the Johns Hopkins University White-Matter Tractography Atlas for which significant decreased AD values were found in CM compared to EM considering the effect of time from onset of CM

White Matter tract	Minimum *p*-value (uncorrected)	Volume (mm^3^)	MNI peak coordinate (mm), (x,y,z)
Corticospinal tract R	.0012	31	(4,-35,-16)

The column Volume represents the volume from the atlas region with significant differences (FDR-corrected and number of contrasts). The maximum significant uncorrected p-value after corrections was .0020. No regions with volume equal or lower than 30 mm^3^ were included in this Table

*R* Right

In the following subsections, the FWE-corrected results for the diverse covariates are shown.

##### Results corrected for duration of migraine history

Including the duration of migraine history as a covariate, no significant differences were found for FA, MD or RD. Significant decreased AD values in CM with respect to EM were found only in the middle cerebellar peduncle (675 mm^3^ with *p* < .05 FWE-corrected, minimum *p*-value = .028), but other 26 regions remained with *p* < .1 FWE-corrected (Additional file 1: Table S3). Also, significant increased AD values in EM with respect to HC were found in seven regions from the left hemisphere (Additional file 1: Table S4). These results can be seen in Additional file 1: Figure S2. No significant differences were found between all migraineurs together and HC, or between CM and HC.

Adding the presence of aura as a covariate, significant increased AD values in EM with respect to HC were found in seven regions from the left hemisphere. These seven regions were the same regions that showed significant differences in the analysis including only the duration of migraine history as a covariate. No significant differences were found for FA, MD or RD.

##### Results corrected for time from onset of CM

In the additional comparisons for CM patients, including time from onset of CM as a covariate, no significant differences were found for MD or RD. Significant decreased AD values in CM with respect to EM were found in 23 regions with *p* < .05 FWE-corrected (Additional file 1: Table S5). Significant decreased FA values in CM compared to HC were found in 15 regions, most of them from the right hemisphere (Additional file 1: Table S6). These results are shown in Additional file 1: Figure S3.

#### Effect size

When comparing between CM and EM Axial Diffusivity values, all Cohen’s d values (except for left fornix) were negative, which means that AD values were lower in CM. For the middle cerebellar peduncle, left external capsule and right sagittal stratum, the Cohen’s d absolute values were equal or greater than .5, a medium effect size according to [45]. A very similar trend, but with lower Cohen’s d absolute values, was found in CM with respect to HC. In the comparison between EM and HC, Cohen’s d value was positive in almost all regions, i.e., AD values were higher in EM with respect to HC. In the pontine crossing tract, a medium effect size was obtained (d = .59). These results are depicted in Additional file 1: Table S7 and Figures S4 and S5. Results for the other DTI measures can be seen in Additional file 1: Figures S6, S7, S8, S9, S10 and S11.

A summary of these results can be seen in Table 6.

**Table 6:** Summary of white matter regions where significant differences were found in all comparisons

White Matter tract	EM > HC*	CM < HC*	CM < EM
Middle cerebellar peduncle	NS	NS	AD
			AD + durM*
			AD + onsCM
Superior cerebellar peduncle R/L	NS	FA + onsCM (R)	AD
			AD + durM* (*p* < 0.1)
			AD + onsCM
Inferior cerebellar peduncle R/L	NS	NS	AD
			AD + durM* (*p* < 0.1)
			AD + onsCM (L)
Superior longitudinal fasciculus R/L	NS	NS	AD
			AD + durM* (*p* < 0.1)
			AD + onsCM* (R)
Genu of corpus callosum	NS	NS	AD
Body of corpus callosum	NS	FA + onsCM	AD
			AD + durM* (*p* < 0.1)
Splenium of corpus callosum	NS	FA + onsCM	AD
			AD + durM* (*p* < 0.1)
			AD + onsCM*
Anterior corona radiata R/L	NS	FA + onsCM (R)	AD
Superior corona radiata R/L	AD + durM (L)	FA + onsCM (R)	AD
			AD + durM* (*p* < 0.1)
			AD + onsCM*
Posterior corona radiata R/L	NS	FA + onsCM (R)	AD
			AD + durM* (L, *p* < 0.1)
			AD + onsCM* (R)
External capsule R/L	AD + durM (L)	FA + onsCM (R)	AD
			AD + durM* (*p* < 0.1)
			AD + onsCM (L; R*)
Posterior limb of internal capsule R/L	AD + durM (L)	FA + onsCM (R)	AD
			AD + durM* (*p* < 0.1)
			AD + onsCM*
Retrolenticular part of internal capsule R/L	AD + durM (L)	FA + onsCM (R)	AD
			AD + durM* (*p* < 0.1)
			AD + onsCM*
Anterior limb of internal capsule R/L	NS	FA + onsCM (R)	AD
Sagittal stratum R/L	AD + durM (L)	FA + onsCM (R)	AD
			AD + durM* (R, *p* < 0.1)
			AD + onsCM* (R)
Posterior thalamic radiation R/L	AD + durM (L)	NS	AD
			AD + durM* (*p* < 0.1)
			AD + onsCM* (R)
Cerebral peduncle R/L	AD + durM (L)	NS	AD
			AD + durM* (*p* < 0.1)
			AD + onsCM*
Corticospinal tract R/L	NS	NS	AD
			AD + durM* (*p* < 0.1)
			AD + onsCM* (R^+^)
Medial lemniscus R/L	NS	NS	AD
Pontine crossing tract	NS	NS	AD
			AD + durM* (*p* < 0.1)
			AD + onsCM
Fornix (cres) R/L	NS	FA + onsCM (R)	AD
			AD + durM* (R)
			AD + onsCM* (R)
Cingulum (hippocampus) L	NS	NS	AD
Anterior thalamic radiation R/L	NS	NS	AD^+^
Forceps major	NS	NS	AD^+^
Inferior longitudinal fasciculus R	NS	NS	AD^+^

The column Volume represents the volume from the atlas region with significant results. No regions with volume equal or lower than 30 mm^3^ were included in this Table

*durM* Duration of migraine as covariate, *L* Left, *NS* Non-significant, *onsCM* Time from onset of CM as covariate, *R* Right; * = only FWE-corrected; ^+^ = significant only in the White-Matter Tractography Atlas

### Correlation analysis

**Fig. 2:**
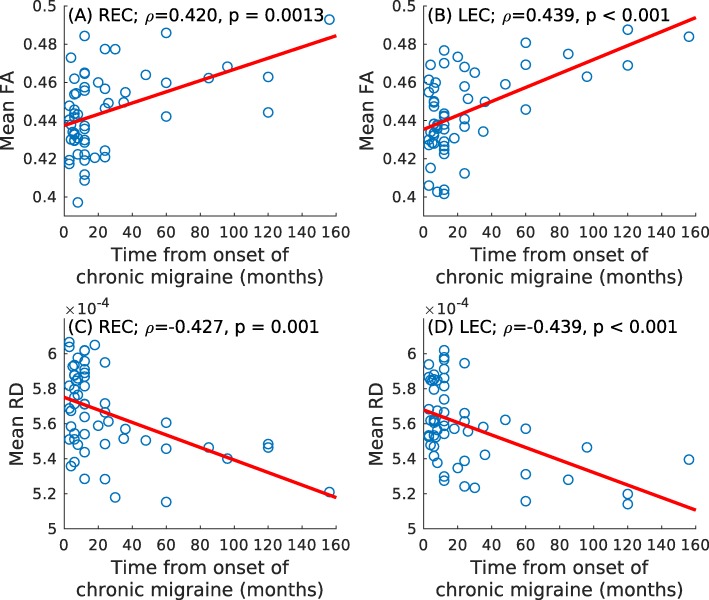
Association graphs between clinical parameters and DTI measures. Significant association with the mean FA in the bilateral external capsule is shown in (**a**) and (**b**). Significant association with the mean RD is shown in (**c**) and (**d**). LEC = left external capsule; REC = right external capsule

After multiple comparisons correction, ROI-based significant positive correlations between time from onset of chronic migraine and mean FA in the right (ρ = .420, *p* = .001) and left (ρ = .439, *p* < .001) external capsule were found. Significant negative correlations between time from onset of chronic migraine and mean RD in the right (ρ = −.427, *p* = .001) and left (ρ = −.439, *p* < .001) external capsule were found. These results can be seen in Fig. 2.

No significant correlations were found neither for mean MD or mean AD, nor duration of the migraine, headache and migraine frequency for both migraine groups in ROI-based correlation analysis.

## Discussion

In a TBSS-based dMRI analysis, in relation with the first stated objective of the study, white matter structural changes in Chronic Migraine compared to Episodic Migraine patients were found in 38 regions when AD was considered as DTI measure. These findings suggest global white matter changes in CM compared to EM.

In the analysis excluding patients with migraine with aura, no new results were observed with respect to the analysis with the whole sample, and the regions with significant results were a subset of the regions of the original analysis. Using only patients without aura, higher *p*-values and lower number of regions with significant differences are obtained. These results suggest that the differences are caused by a loss of statistical power more than the effect of aura itself. Including duration of migraine history as a covariate, differences were found between EM and Healthy Controls in seven regions, but they did not survive the FDR and the number of contrasts corrections. The addition of the presence of aura as a covariate did not change the results with respect to using only the duration of migraine history as a covariate.

Additionally, including time from onset of chronic migraine as a covariate in the comparisons with CM patients, significant decreased values were found in AD between CM and EM. Decreased values were also found in FA in CM with respect to HC, but they did not survive the FDR and the number of contrasts corrections.

In relation with the second objective of the study, correlation analysis was executed between diverse DTI measures and clinical features. Significant correlations between time from onset of chronic migraine and mean FA (positive correlation) and mean RD (negative correlation) in the bilateral external capsule were found.

White matter differences between both groups of migraineurs were not previously found by Neeb et al. in the only study, to the best of our knowledge, which assessed patients with chronic and episodic migraine using DTI [22]. In the present study, however, a considerably bigger cohort was included when compared to that study, and our participants were considerably younger. The influence of age in the white matter diffusion is well-known and has been extensively assessed. The most common pattern is to find decreased FA and increased RD values in older people, while the AD pattern is unclear [30, 46]. Considering the aging-effect, altered DTI measures in healthy people might reduce the diffusion differences between patients and controls in older subjects.

Differences between the two migraine groups were found using AD as a DTI measure. It is known, however, that relationships between DTI-derived parameter changes and specific microstructure alterations are difficult to establish, and therefore results must be interpreted carefully. Winklewski et al. interpreted reduced AD values, as we found in CM compared to EM, as the beginning of demyelination [47]. This reduction, nevertheless, could be ineffective to detect prolonged demyelination [47]. Nonetheless, based on studies in mice, a review study by Alexander et al. linked AD more to axonal damage than demyelination [48]. In a posterior study also in mice, Sun et al. established a relationship between reduced AD values and axonal damage, and between increased RD values and myelin damage, confirmed with immunohistochemistry examinations [49]. In a human study, Pierpaoli et al. found decreased AD in primary lesions and in regions with secondary white matter degeneration [50]. This axonal loss hypothesis has also been exposed for Alzheimer’s disease [51] and migraine patients [7].

Previous whole-brain TBSS studies found white matter differences between migraine patients and healthy controls [6–11, 52]. Most of these studies report decreased FA in migraine patients with respect to healthy controls, but one study showed increased FA in migraineurs with respect to healthy controls; in this study, Messina et al. analysed paediatric patients [9], which could explain the difference. Decreased FA can be caused by factors like demyelination, lower packing density or different membrane permeability [53] and is modulated by characteristics such as axon diameter and packing or fibre organization [54].

Results for MD and RD in the literature regarding migraine using TBSS are unclear. On the one hand, increased MD and RD values in migraineurs with respect to healthy controls were obtained in [11, 52], but on the other hand, decreased values were reported in [7–9]. Results obtained with methods different than TBSS, such as ROI-based analysis or tractography, showed increased MD and/or RD values in migraine patients with respect to healthy controls [13, 14, 18, 55]. Increased RD could also be a biomarker of demyelination [47, 48]. There are also studies that found no differences between migraine patients and healthy controls. These studies employed methods whole-brain such as TBSS [22, 56], ROI-based analysis (ictal migraine) [57] and voxel-based whole brain comparison [58].

Interestingly, the trend when comparing AD values between EM and healthy controls is inverted when comparing CM and healthy controls or CM with respect to EM (Additional file 1: Figures S4 and S5). As previously mentioned, AD might be an indicator of axonal loss. This might indicate that the evolution from EM to CM is characterised by a loss of axonal integrity. This result also shows that, in migraine, there could be different processes of axonal behaviour involving different pathophysiological mechanisms.

In contrast to our results, some TBSS studies obtained decreased AD in migraine patients with respect to healthy controls [7–9]. Petrušić et al. also found decreased AD in migraine with aura patients with respect to healthy controls using a tractography approach [15]. We detected the same trend, but only in CM patients. We obtained the opposite result in EM patients, when including duration of migraine as a covariate, but this result was not significant in the post-hoc analysis. In [7, 8], cohort differences (considering only the EM patients in our case), i.e., lower disease duration and higher attack frequency, with possibly high frequency EM patients in the sample of [7, 8], could explain the differences between the studies. In [9], as previously stated, paediatric patients were included in the sample by Messina et al., which could explain the difference.

The analysis of the temporal change in migraine patients adds an interesting insight to the former results.

In the case of EM patients, considering the duration of migraine as a covariate, there was a lower number of regions with significant differences with respect to CM patients. The significant difference in the duration of migraine between both groups of migraine patients could be a confounding factor in the previous results. Furthermore, significant increased AD values in EM compared to HC were observed, possibly due to more precise estimations (less variability) in the case of the values in EM patients.

These results seem to indicate a temporal evolution in CM patients that reflects an adaptation to continuous headache attacks. In the initial months with CM (“short-term” patients), axonal integrity seems to be damaged, as suggested by decreased FA values in those patients compared to HC when including time from onset of CM as a covariate. Decreased FA values in CM patients with therapy compared to HC, after 6 months follow-up, were reported previously by Gomez-Beldarrain et al. [16]. Considering these results and the decreased AD values in CM with respect to EM, it seems that in progression from EM to CM there might be a process that causes severe white matter alterations.

Later, in CM patients, a set of plastic changes as an adaptation to the frequent headaches may happen. The white matter reorganisation is suggested by positive correlation between FA and time from onset of CM, and by the simultaneous negative correlation between RD and time from onset of CM.

In line with our CM correlation results, Szabó et al., 2017 [10], obtained a trend showing increased FA in migraine with aura patients with respect to healthy controls. In this study, based on increased FA values, the authors hypothesised that repeated painful conditions or increased cortical excitability might cause maladaptive plastic changes in migraine with aura. Moreover, increased FA values were found in people with repeated stimuli in learning processes [59, 60], so something similar could be happening in CM patients, who suffer repeated painful stimuli.

Regarding correlation analysis, we obtained significant correlations between time from onset of chronic migraine and DTI measures (FA and RD) in the bilateral external capsule, but no significant correlations were obtained with the duration of migraine in CM or EM patients.

The external capsule is a part of the central core, a network on top of the brainstem that includes structures like the insular surface, the extreme and internal capsules, the lentiform nucleus or the thalamus [61]. The extreme and external capsules lie in anteroposterior disposition, and they are connected to the anteroinferior part of the insula [61]. The lentiform nucleus is located between the external and internal capsules [61]. The internal, external and extreme capsules connect the insular surface, basal ganglia and thalamus to the cerebral lobes [61]. Russo et al. reported that, in the insula and lentiform nucleus, migraine patients, compared to HC, are characterised by an increased blood oxygenation level dependent response [62]. Furthermore, Borsook et al. exposed that the insula is implicated in processes related to the clinical presentation of migraine and is a “hub of activity” in migraine [63]. The role of the external capsule in anteroposterior connections and in connections between subcortical regions implied in migraine and cortical regions could be highly relevant in migraine pathogenesis, especially in Chronic Migraine.

In other studies, significant negative correlations between duration of migraine in years (in EM patients) and FA [5, 7], MD [7] or AD [7, 10] were obtained, but also significant positive correlation between duration of migraine and MD [14]. This discrepancy could be explained with methodological and cohort differences. No significant correlation between DTI measures and the external capsule has been previously found in migraine patients, but no correlation between time from onset of chronic migraine and DTI measures has been previously assessed.

In this study, high frequency EM patients (10–14 headache days per month) were excluded. This decision was made in order to avoid misclassified patients, which could mislead the analysis [28]. Compared to Neeb et al. [22], CM patients from our sample had greater headache frequency, while EM patients from our sample had lower headache frequency. This increased difference between the migraine groups, together with the larger cohort size, could be a factor explaining why we obtained significant results in the CM-EM comparison, while no significant results were obtained in [22]. However, no significant correlations were found between headache frequency and DTI measures, which could mean that headache frequency does not have a very relevant effect on diffusion within the EM or CM groups. In any case, a deeper specific analysis, focusing on high frequency EM patients, would be needed to clarify whether this group of patients is closer to the low frequency EM group or to the CM group.

There are several strengths and limitations in this study. About the strengths, this study is, to the best of our knowledge, the white matter study with the highest number of participants simultaneously including Healthy Controls, Episodic Migraine and Chronic Migraine patients. Moreover, the selection criteria of the patients allowed us to detect significant differences not found previously, especially between EM and CM patients.

About the limitations of this study, due to time constraints in the MRI acquisition process in a clinical setting, we acquired no T2 or T2-FLAIR MRI sequences that would be helpful to assess White Matter Hyperintensities (WMHs). Migraine has been associated with an increased risk for WMHs detected on MRI [64]; also pain in EM patients [65] and an unfavourable prognosis [66] were found to be associated with the occurrence of WMHs. Considering our correlation results in CM patients and the state of the art, the WMHs analysis would have been interesting in this study. Medication overuse was identified in an important percentage of the CM patients in our sample (75%). This might be a confounding factor, due to possible structural differences in the white matter with respect to CM patients without overuse. The exclusion of patients with anxiety or depression implies that there was no chance to assess possible effects of these conditions on brain structure in migraine patients. Anxiety and depression are often comorbid in patients with migraine [67–69]. When MRI were acquired in the patients, they had no attacks in the previous 24 h, but they could be in a prodromal stage, as we did control time from past, but not to the next migraine attack. Altered brain physiology and abnormal functional connectivity have been found in prodromal stages [70, 71], so this is a possible source of bias in the results. Diagnosis of infrequent TTH in controls was done solely by history and not by using a headache diary; however, they were excluded if other headache disorders were present or the frequency of headache in the preceding year was > 1 headache day per month or > 12 headache days per year. Finally, in the analysis of the presence of aura, the number of patients with migraine with aura was too small to additionally compare the changes in migraine with aura against migraine without aura.

**Fig. 3:**
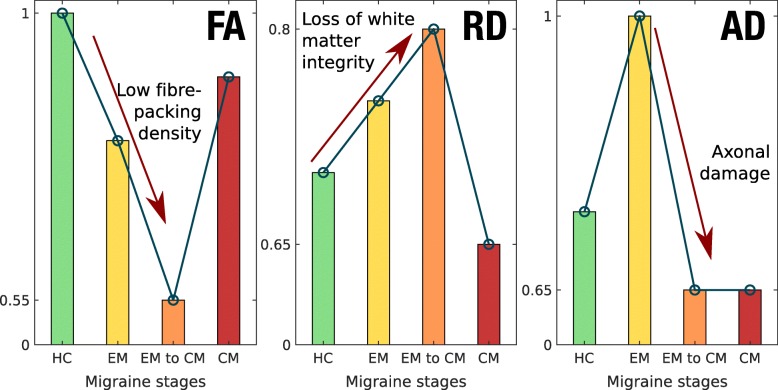
DTI measures temporal change hypothesis. Illustrative values are shown for generalized trends in FA, RD and AD (from left to right) in each of the different migraine stages, including a previous healthy control stage. Stages are ordered chronologically from left to right in each subplot. The interpretation of different trends in DTI measures is given in each subplot. The values in the vertical axes should only be used as an orientation to watch the trends and differences between groups, not interpreted as real values

In summary, considering previous studies and our results, a hypothesis about the migraine process could be drafted that distinguishes three states or stages: EM, transition from EM to CM, and CM. In the EM state, there would be some white matter damage, produced mainly by a loss of axonal integrity. Then, in the transition from EM to CM, there would be a loss of axonal integrity, but probably not led by a severe additional damage in myelin. Finally, in the CM state, there would be a series of plastic changes, as an adaptation to a continuous ictal state. Considering this evolution hypothesis, EM might involve a coexistence of loss of white matter integrity and maladaptive plasticity, with more severe integrity damage in the transition to CM, and CM may show predominant maladaptive plasticity, being, in this regard, a different entity with respect to a more frequent EM. An illustration of the hypothesised temporal evolution of the three main DTI measures used here can be seen in Fig. 3.

Anyway, regarding this hypothesis, longitudinal analysis should be performed in order to confirm this possible evolution, and the interpretation of the DTI measures must be carried out cautiously, as mentioned before in this section. Additionally, about the transition from EM to CM, the high frequency EM patients could be especially interesting to investigate. Indeed, it remains to be elucidated whether high frequency EM is actually a transition phase between EM and CM, an intense EM or, considering an extreme case, a low frequency CM.

Although this can be only considered a preliminary hypothesis, it would contribute to explain the high variability in the results in the literature found when comparing migraineurs with healthy controls in terms of white matter diffusion parameters. Indeed, if different trends can be found at different stages of the disease, then the results of global comparisons would depend heavily on the internal composition of the cohort of migraine patients in each study, possibly yielding what seem to be opposite results.

## Conclusions

The current findings suggest global white matter structural differences between Episodic Migraine and Chronic Migraine, with damaged axonal integrity in Chronic Migraine, and between both groups of migraine patients and Healthy Controls. A different temporal pathophysiological evolution with maladaptive plastic changes seems to happen in Chronic Migraine with respect to Episodic Migraine. Further research is needed for these findings to be confirmed. Also, additional clinical features should be considered, longitudinal evaluation should be performed and a possible relationship with functional changes should be assessed.

## Supplementary information

**Additional file 1: Figure S1.** White matter alterations in chronic migraine compared to episodic migraine patients. **Table S1.** White matter regions where decreased AD values were found in CM compared to EM (FWE-corrected). **Table S2.** White matter regions where decreased AD values were found in CM compared to EM considering only patients with migraine without aura (FWE-corrected). **Table S3.** White matter regions from the ICBM-DTI-81 White Matter Atlas for which decreased AD values were found in CM compared to EM considering the effect of duration of migraine history (FWE-corrected). **Table S4.** White matter regions from the ICBM-DTI-81 White Matter Atlas for which increased AD values were found in EM compared to HC considering the effect of duration of migraine history (FWE-corrected). **Figure S2.** White matter alterations in migraine including duration of migraine history as a covariate. **Table S5.** White matter regions from the ICBM-DTI-81 White Matter Atlas for which significant AD values were found in CM compared to EM considering the effect of time from onset of CM (FWE-corrected). **Table S6.** White matter regions from the ICBM-DTI-81 White Matter Atlas for which decreased FA values were found in CM compared to HC considering the effect of time from onset of CM (FWE-corrected). **Figure S3.** White matter alterations in CM including time from onset of chronic migraine as a covariate. **Table S7**. Cohen’s d skeleton AD values in regions with significant differences between EM and CM. **Figure S4.** Cohen’s AD bar plots of regions with FWE-corrected differences between EM and CM (part 1). **Figure S5.** Cohen’s AD bar plots of regions with FWE-corrected differences between EM and CM (part 2). **Figure S6.** Cohen’s d FA bar plots (part 1). **Figure S7**. Cohen’s d FA bar plots (part 2). **Figure S8.** Cohen’s d RD bar plots (part 1). **Figure S9.** Cohen’s d RD bar plots (part 2). **Figure S10.** Cohen’s d MD bar plots (part 1). **Figure S11.** Cohen’s d MD bar plots (part 2).

## Data Availability

All patients who participated in the study signed a written informed consent. This consent did not include a statement to make the individual confidential data accessible to the public. Therefore, anonymized data are available from the corresponding author on reasonable request.

## Abbreviations

AD: Axial diffusivity
ANCOVA: Analysis of covariance
BET: Brain extraction tool
CM: Chronic migraine
dMRI: Diffusion magnetic resonance imaging
DTI: Diffusion tensor imaging
DWI: Diffusion-weighted image
EM: Episodic migraine
FA: Fractional anisotropy
FDR: False discovery rate
fMRI: Functional magnetic resonance imaging
FWE: Family wise error
HC: Healthy controls
ICHD-3: International classification of headache disorders third edition
MD: Mean diffusivity
MNI: Montreal neurological institute
MRI: Magnetic Resonance Imaging
RD: Radial diffusivity
ROI: Region of interest
TBSS: Tract-based spatial statistics
TE: Echo time
TFCE: Threshold-free cluster enhancement
TFE: Turbo field echo
TR: Repetition time
TTH: Tension-type headache
