# Supplementary material for: White matter changes in chronic and episodic migraine: a diffusion tensor imaging study
Source: J Headache Pain. 2020 Jan 2;21(1):1. doi: 10.1186/s10194-019-1071-3 (PMC6941267; doi:10.1186/s10194-019-1071-3)
Supplement: Supplementary file 1 — Additional file 1: Figure S1. White matter alterations in chronic migraine compared to episodic migraine patients. Table S1. White matter regions where decreased AD values were found in CM compared to EM (FWE-corrected). Table S2. White matter regions where decreased AD values were found in CM compared to EM considering only patients with migraine without aura (FWE-corrected). Table S3. White matter regions from the ICBM-DTI-81 White Matter Atlas for which decreased AD values were found in CM compared to EM considering the effect of duration of migraine history (FWE-corrected). Table S4. White matter regions from the ICBM-DTI-81 White Matter Atlas for which increased AD values were found in EM compared to HC considering the effect of duration of migraine history (FWE-corrected). Figure S2. White matter alterations in migraine including duration of migraine history as a covariate. Table S5. White matter regions from the ICBM-DTI-81 White Matter Atlas for which significant AD values were found in CM compared to EM considering the effect of time from onset of CM (FWE-corrected). Table S6. White matter regions from the ICBM-DTI-81 White Matter Atlas for which decreased FA values were found in CM compared to HC considering the effect of time from onset of CM (FWE-corrected). Figure S3. White matter alterations in CM including time from onset of chronic migraine as a covariate. Table S7. Cohen’s d skeleton AD values in regions with significant differences between EM and CM. Figure S4. Cohen’s AD bar plots of regions with FWE-corrected differences between EM and CM (part 1). Figure S5. Cohen’s AD bar plots of regions with FWE-corrected differences between EM and CM (part 2). Figure S6. Cohen’s d FA bar plots (part 1). Figure S7. Cohen’s d FA bar plots (part 2). Figure S8. Cohen’s d RD bar plots (part 1). Figure S9. Cohen’s d RD bar plots (part 2). Figure S10. Cohen’s d MD bar plots (part 1). Figure S11. Cohen’s d MD bar plots (part 2). [file 10194_2019_1071_MOESM1_ESM.pdf]

Supplementary material for:

# White Matter Changes in Chronic and Episodic Migraine: A Diffusion Tensor Imaging Study

Álvaro Planchuelo-Gómez[1], David García-Azorín[2], Ángel L. Guerrero[2,3,4*], Santiago Aja-Fernández[1], Margarita Rodríguez[5] and Rodrigo de Luis-García[1]

Corresponding author: Dr Ángel L. Guerrero

Email: gueneurol@gmail.com

Supplementary Figure 1: White matter alterations in chronic migraine compared to episodic migraine patients

Supplementary Figure 2: White matter alterations in migraine including duration of migraine history as a covariate

Supplementary Figure 3: White matter alterations in chronic migraine including time from onset of chronic migraine as a covariate

Supplementary Figure 4: Cohen's AD bar plots of regions with FWE-corrected differences between EM and CM (part 1)

Supplementary Figure 5: Cohen's AD bar plots of regions with FWE-corrected differences between EM and CM (part 2)

Supplementary Figure 6: Cohen's d FA bar plots (part 1)

Supplementary Figure 7: Cohen's d FA bar plots (part 2)

Supplementary Figure 8: Cohen's d RD bar plots (part 1)

Supplementary Figure 9: Cohen’s d RD bar plots (part 2)

Supplementary Figure 10: Cohen’s d MD bar plots (part 1)

Supplementary Figure 11: Cohen’s d MD bar plots (part 2)

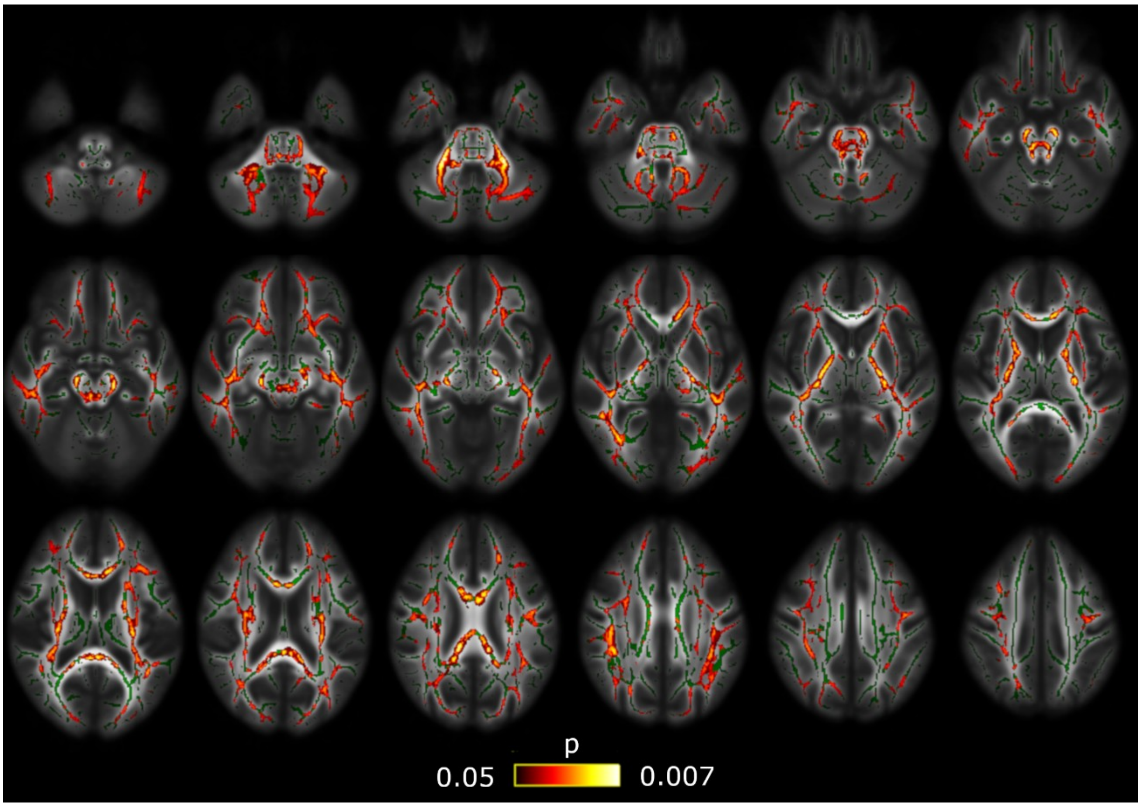


**Supplementary Figure 1. White matter alterations in chronic migraine compared to episodic migraine patients.** TBSS shows decreased AD values in CM compared to EM in widespread locations. White matter skeleton is shown in green, and voxels with significant differences in red-yellow. The colour bar shows the p-values (FWE-corrected).

**Supplementary Table 1**. White matter regions where decreased AD values were found in CM compared to EM (FWE-corrected).

| White Matter tract | Minimum p-value (FWE-corrected) | Volume ($mm^3$) | MNI peak coordinate (mm), (x,y,z) |
|---|---|---|---|
| Middle cerebellar peduncle | .007 | 2206 | (-20,-50,-32) |
| Superior cerebellar peduncle R/L | .020/.020 | 142/126 | (5,-28,-19) / (-4,-28,-19) |
| Inferior cerebellar peduncle R/L | .019/.009 | 75/89 | (12,-43,-35) / (-13,-45,-31) |
| Superior longitudinal fasciculus R/L | .021/.021 | 971/874 | (33,-4,20) / (-36,-49,15) |
| Genu of corpus callosum | .019 | 455 | (-10,28,1) |
| Body of corpus callosum | .032 | 842 | (-4,-30,23) |
| Splenium of corpus callosum | .025 | 873 | (22,-50,25) |
| Anterior corona radiata R/L | .024/.018 | 556/805 | (18,21,-11) / (-18,38,-1) |
| Superior corona radiata R/L | .020/.022 | 666/396 | (28,-16,21) / (-27,-11,20) |
| Posterior corona radiata R/L | .022/.022 | 201/214 | (25,-24,24) / (-30,-52,22) |
| External capsule R/L | .020/.018 | 459/695 | (30,-10,14) / (-22,16,-12) |
| Posterior limb of internal capsule R/L | .020/.022 | 569/536 | (26,-17,13) / (-27,-17,17) |
| Retrolenticular part of internal capsule R/L | .023/.023 | 457/344 | (31,-34,15) / (-25,-22,3) |
| Anterior limb of internal capsule R/L | .022/.020 | 216/290 | (15,-1,7) / (-20,18,3) |
| Sagittal stratum R/L | .022/.022 | 471/359 | (37,-49,-4) / (-41,-18,-13) |
| Posterior thalamic radiation R/L | .022/.022 | 353/279 | (37,-50,-2) / (-35,-52,13) |

| | | | |
|---|---|---|---|
| Cerebral peduncle R/L | .020/.022 | 234/265 | (11,-23,-21) / (-9,-19,-20) |
| Corticospinal tract R/L | .019/.023 | 106/165 | (10,-27,-26) / (-7,-18,-22) |
| Medial lemniscus R/L | .020/.015 | 82/103 | (8,-39,-40) / (-7,-37,-40) |
| Pontine crossing tract | .018 | 82 | (8,-31,-27) |
| Fornix (cres) R/L | .024/.024 | 74/45 | (35,-12,-14) / (-34,-15,-13) |
| Cingulum (hippocampus) L | .036 | 56 | (-17,-42,-2) |

FWE = Family-wise error; L = left; R = right.

The column Volume represents the volume from the atlas region with $p < .05$ (FWE-corrected). No regions with volume equal or lower than 30 $mm^3$ were included in this Table.

**Supplementary Table 2**. White matter regions where decreased AD values were found in CM compared to EM considering only patients with migraine without aura (FWE-corrected).

| White Matter tract | Minimum p-value (FWE-corrected) | Volume ($mm^3$) | MNI peak coordinate (mm), (x,y,z) |
|---|---|---|---|
| Middle cerebellar peduncle | .014 | 1382 | (-23,-46,-36) |
| Superior longitudinal fasciculus R | .040 | 388 | (33,-4,20) |
| Superior corona radiata R | .031 | 166 | (28,-17,23) |
| External capsule R | .034 | 103 | (29,-8,18) |
| Posterior limb of internal capsule R | .030 | 428 | (26,-17,13) |
| Retrolenticular part of internal capsule R | .034 | 323 | (31,-30,7) |
| Anterior limb of internal capsule R | .037 | 96 | (15,-1,7) |
| Sagittal stratum R | .034 | 380 | (37,-22,-7) |
| Posterior thalamic radiation R | .037 | 242 | (30,-39,16) |
| Fornix (cres) R | .034 | 52 | (35,-16,-12) |

FWE = Family-wise error; R = right.

The column Volume represents the volume from the atlas region with $p < .05$ (FWE-corrected). No regions with volume equal or lower than 30 $mm^3$ were included in this Table.

**Supplementary Table 3**. White matter regions from the ICBM-DTI-81 White Matter Atlas for which decreased AD values were found in CM compared to EM considering the effect of duration of migraine history (FWE-corrected).

| White Matter tract | Minimum p-value (FWE-corrected) | Volume ($mm^3$) | MNI peak coordinate (mm), (x,y,z) |
|---|---|---|---|
| Middle cerebellar peduncle | .028 | 1816 | (-20,-50,-32) |
| Superior cerebellar peduncle R/L | .066/.067 | 138/124 | (5,-28,-19) / (-4,-28,-19) |
| Inferior cerebellar peduncle R/L | .069/.039 | 44/61 | (13,-42,-36) / (-13,-45,-31) |
| Superior longitudinal fasciculus R/L | .088/.079 | 254/411 | (42,-43,3) / (-36,-50,15) |
| Body of corpus callosum | .094 | 233 | (13,-29,28) |
| Splenium of corpus callosum | .090 | 618 | (-10,-33,25) |
| Superior corona radiata R/L | .091/.095 | 261/71 | (28,-15,21) / (-27,-11,20) |
| Posterior corona radiata L | .080 | 116 | (-28,-51,25) |
| External capsule R/L | .076/.095 | 94/63 | (35,-14,-9) / (-28,-10,17) |
| Posterior limb of internal capsule R/L | .087/.095 | 77/126 | (26,-17,13) / (-23,-20,13) |
| Retrolenticular part of internal capsule R/L | .078/.095 | 323/81 | (38,-29,-1) / (-29,-38,14) |
| Sagittal stratum R | .066 | 407 | (37,-49,-3) |
| Posterior thalamic radiation R/L | .066/.081 | 256/54 | (37,-50,-3) / (-35,-52,13) |
| Cerebral peduncle R/L | .068/.092 | 153/127 | (9,-19,-21) / (-9,-19,-20) |
| Corticospinal tract R/L | .067/.093 | 98/109 | (8,-27,-26) / (-7,-18,-22) |
| Pontine crossing tract | .067 | 51 | (8,-31,-27) |

| | | | |
|---|---|---|---|
| Fornix (cres) R | .024 | 37 | (35,-12,-14) |

FWE = Family-wise error; L = left; R = right.

The column Volume represents the volume from the atlas region with p-values < .1 (FWE-corrected). No regions with volume equal or lower than 30 $mm^3$ were included in this Table.

**Supplementary Table 4**. White matter regions from the ICBM-DTI-81 White Matter Atlas for which increased AD values were found in EM compared to HC considering the effect of duration of migraine history (FWE-corrected).

| White Matter tract | Minimum p-value (FWE-corrected) | Volume ($mm^3$) | MNI peak coordinate (mm), (x,y,z) |
|---|---|---|---|
| Superior corona radiata L | .026 | 116 | (-26,-11,20) |
| External capsule L | .022 | 303 | (-34,-15,-8) |
| Posterior limb of internal capsule L | .024 | 472 | (-15,-10,0) |
| Retrolenticular part of internal capsule L | .025 | 347 | (-33,-34,6) |
| Sagittal stratum L | .023 | 133 | (-36,-17,-9) |
| Posterior thalamic radiation L | .025 | 38 | (-35,-39,7) |
| Cerebral peduncle L | .024 | 105 | (-15,-13,-5) |

FWE = Family-wise error; L = left.

The column Volume represents the volume from the atlas region with p-values < .05 (FWE-corrected). No regions with volume equal or lower than 30 $mm^3$ were included in this Table.

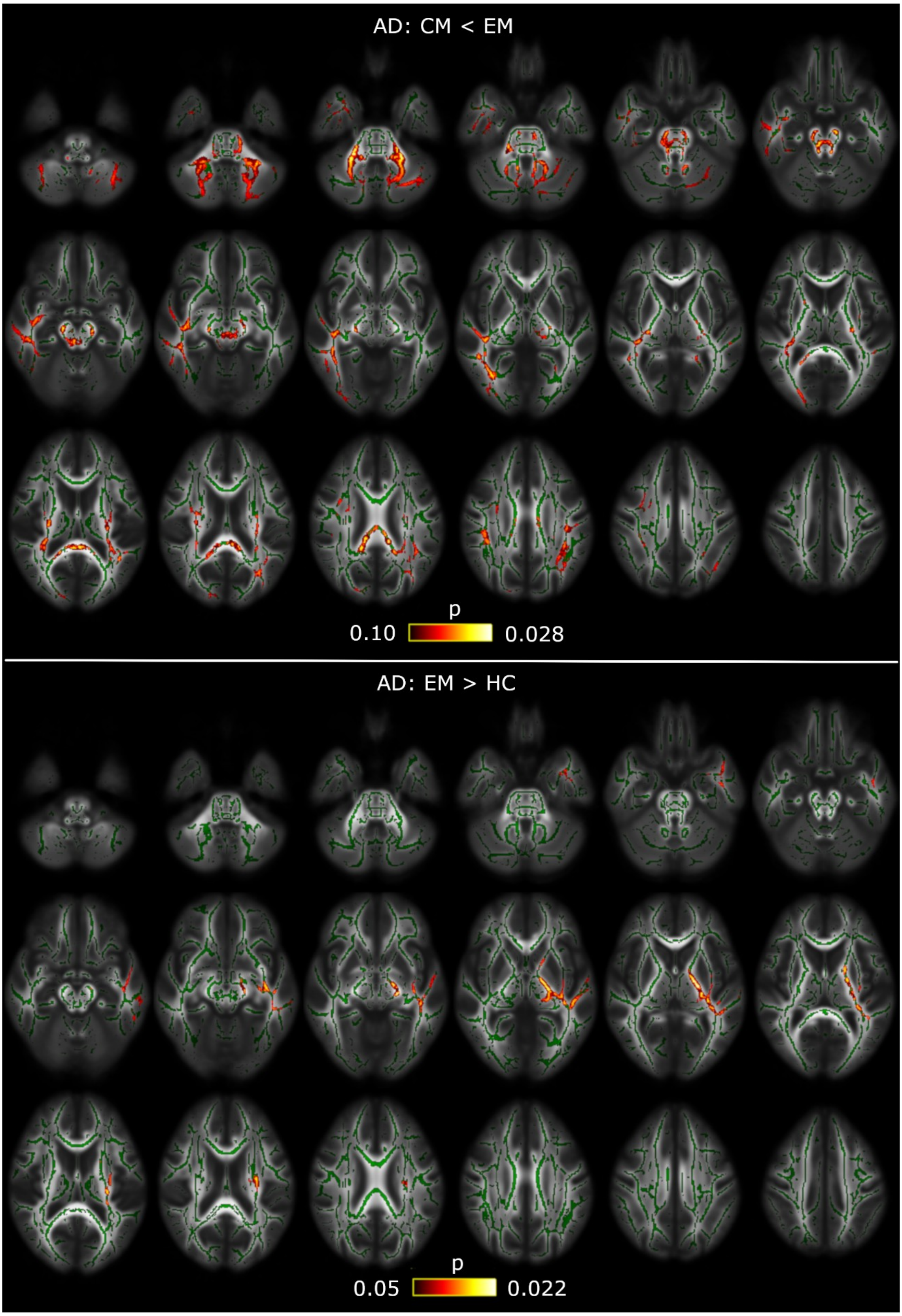

AD: CM < EM
p
0.10
0.028
AD: EM > HC
p
0.05
0.022

**Supplementary Figure 2. White matter alterations in migraine including duration of migraine history as a covariate**. TBSS showed decreased AD values in CM compared to EM in widespread locations (top) and increased AD values in EM compared to healthy controls (bottom) in left hemisphere locations. White matter skeleton is shown in green, and voxels with the lowest p-values in red-yellow. The colour bar shows the p-values (FWE-corrected).

**Supplementary Table 5**. White matter regions from the ICBM-DTI-81 White Matter Atlas for which significant AD values were found in CM compared to EM considering the effect of time from onset of CM (FWE-corrected).

| White Matter tract | Minimum p-value (FWE-corrected) | Volume ($mm^3$) | MNI peak coordinate (mm), (x,y,z) |
|---|---|---|---|
| Middle cerebellar peduncle | .023 | 1295 | (-20,-50,-32) |
| Superior cerebellar peduncle R/L | .028/.030 | 101/115 | (5,-28,-19) / (-4,-28,-19) |
| Inferior cerebellar peduncle L | .035 | 46 | (-10,-50,-25) |
| Superior longitudinal fasciculus R | .033 | 720 | (32,-4,20) |
| Splenium of corpus callosum | .042 | 32 | (18,-49,27) |
| Superior corona radiata R/L | .030/.032 | 335/136 | (28,-15,19) / (-27,-11,20) |
| Posterior corona radiata R | .035 | 83 | (28,-34,19) |
| External capsule R/L | .033/.031 | 127/171 | (30,-10,14) / (-28,-10,18) |
| Posterior limb of internal capsule R/L | .029/.032 | 301/281 | (26,-17,13) / (-27,-17,17) |
| Retrolenticular part of internal capsule R/L | .031/.032 | 473/59 | (30,-29,7) / (-25,-23,12) |
| Sagittal stratum R | .034 | 150 | (38,-28,-4) |
| Posterior thalamic radiation R | .034 | 51 | (30,-39,16) |
| Cerebral peduncle R/L | .028/.031 | 207/258 | (10,-28,-16) / (-9,-20,-20) |
| Corticospinal tract R/L | .030/.031 | 105/125 | (11,-22,-22) / (-7,-18,-22) |
| Pontine crossing tract | .030 | 56 | (4,-26,-24) |
| Fornix (cres) R | .037 | 46 | (35,-12,-14) |

FWE = Family-wise error; L = left; R = right.

The column Volume represents the volume from the atlas region with p-values < .05 (FWE-corrected). No regions with volume equal or lower than 30 $mm^3$ were included in this Table.

**Supplementary Table 6**. White matter regions from the ICBM-DTI-81 White Matter Atlas for which decreased FA values were found in CM compared to HC considering the effect of time from onset of CM (FWE-corrected).

| White Matter tract | Minimum p-value (FWE-corrected) | Volume ($mm^3$) | MNI peak coordinate (mm), (x,y,z) |
|---|---|---|---|
| Superior longitudinal fasciculus R | .033 | 271 | (32,-4,19) |
| Body of corpus callosum | .042 | 47 | (19,-30,31) |
| Splenium of corpus callosum | .042 | 209 | (19,-31,31) |
| Anterior corona radiata R | .033 | 295 | (25,17,12) |
| Superior corona radiata R | .038 | 236 | (32,-6,20) |
| Posterior corona radiata R | .039 | 133 | (25,-23,22) |
| External capsule R | .031 | 661 | (30,1,13) |
| Posterior limb of internal capsule R | .039 | 217 | (24,-19,1) |
| Retrolenticular part of internal capsule R | .036 | 425 | (38,-29,0) |
| Anterior limb of internal capsule R | .035 | 83 | (23,17,11) |
| Sagittal stratum R | .036 | 132 | (33,-23,-4) |
| Fornix (cres) R | .035 | 36 | (33,-22,-6) |

FWE = Family-wise error; L = left; R = right.

The column Volume represents the volume from the atlas region with p-values < .05 (FWE-corrected). No regions with volume equal or lower than 30 $mm^3$ were included in this Table.

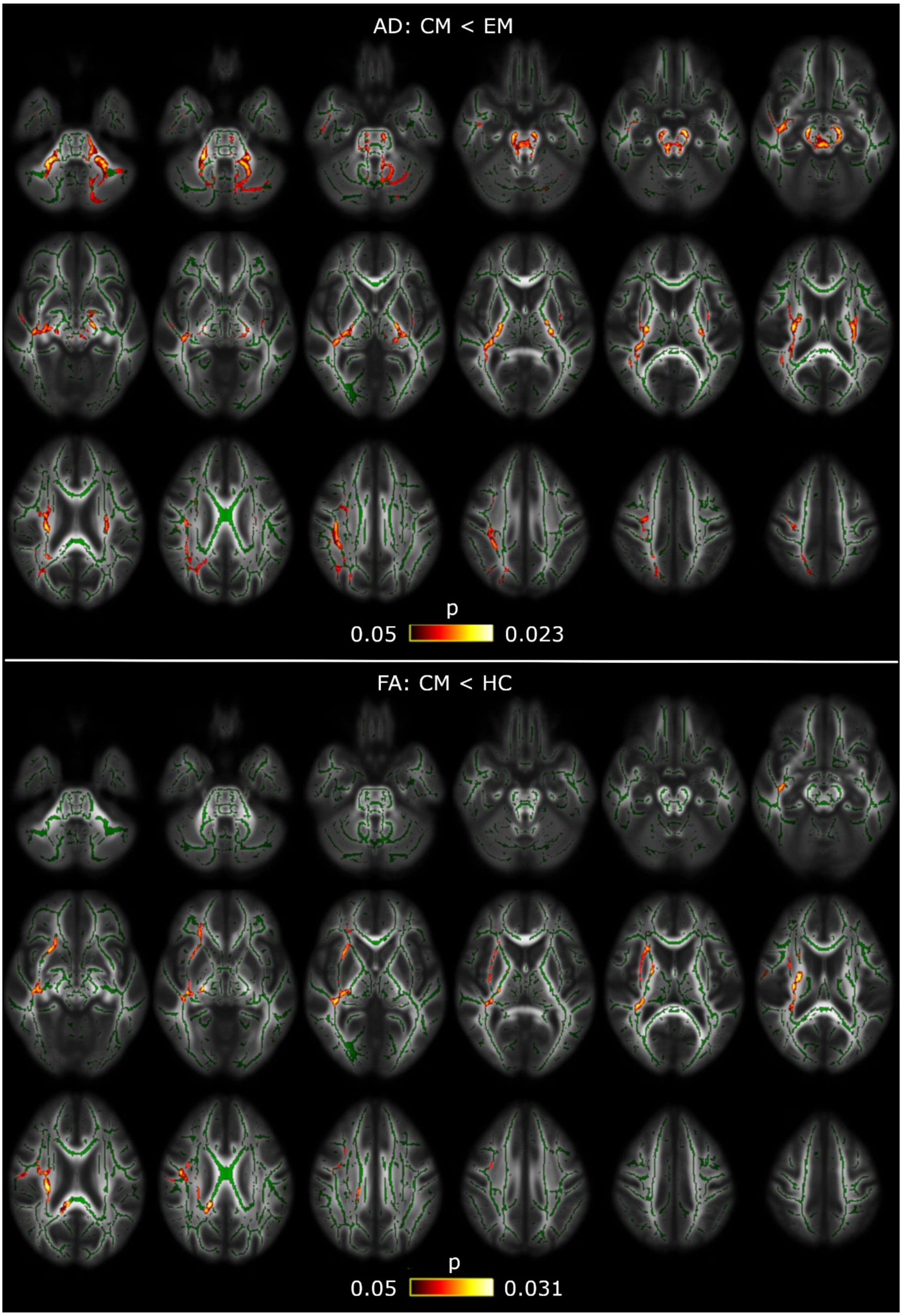
AD: CM < EM
p
0.05
0.023
FA: CM < HC
p
0.05
0.031

**Supplementary Figure 3. White matter alterations in CM including time from onset of chronic migraine as a covariate**. TBSS showed decreased AD values in CM compared to EM in widespread locations (top) and decreased FA values in CM compared to healthy controls (bottom). Mean white matter skeleton is shown in green, and voxels with the lowest p-values in red-yellow. The colour bar shows the p-values (FWE-corrected).

**Supplementary Table 7**. Cohen's d skeleton AD values in regions with significant differences between EM and CM.

| White matter region | EM vs. HC Cohen's d | CM vs. HC Cohen's d | CM vs. EM Cohen's d |
|---|---|---|---|
| Middle cerebellar peduncle (MCP) | .31 | -.29 | **-.60** |
| Superior cerebellar peduncle R (RSCP)/L (LSCP) | .18/.03 | -.23/-.32 | -.40/-.37 |
| Inferior cerebellar peduncle R (RICP)/L (LICP) | .45/.45 | .21/.16 | -.22/-.28 |
| Superior longitudinal fasciculus R (RSLF)/L (LSLF) | .36/.47 | -.05/.01 | -.39/-.46 |
| Genu of corpus callosum (gCC) | -.01 | -.18 | -.17 |
| Body of corpus callosum (bCC) | .01 | -.09 | -.10 |
| Splenium of corpus callosum (sCC) | -.02 | -.30 | -.29 |
| Anterior corona radiata R (RACR)/L (LACR) | .06/.17 | -.14/-.18 | -.21/-.39 |
| Superior corona radiata R (RSCR)/L (LSCR) | .18/-.07 | -.18/-.19 | -.37/-.13 |
| Posterior corona radiata R (RPCR)/L (LPCR) | .15/-.01 | .01/-.16 | -.13/-.15 |
| External capsule R (REC)/L (LEC) | .17/.41 | -.09/-.05 | -.29/**-.50** |
| Posterior limb of internal capsule R (RPIC)/L (LPIC) | .07/.10 | -.29/-.19 | -.41/-.33 |
| Retrolenticular part of internal capsule R (RRIC)/L (LRIC) | .28/.32 | -.09/.05 | -.39/-.34 |
| Anterior limb of internal capsule R (RAIC)/L (LAIC) | -.07/.23 | -.27/-.01 | -.22/-.29 |
| Sagittal stratum R (RSS)/L (LSS) | .26/.31 | -.30/-.04 | **-.57**/-.36 |
| Posterior thalamic radiation R (RPTR)/L (LPTR) | .07/.06 | -.18/-.12 | -.24/-.18 |
| Cerebral peduncle R (RCP)/L (LCP) | .08/.07 | -.15/-.26 | -.27/-.40 |

| | | | |
|---|---|---|---|
| Corticospinal tract R (RCT)/L (LCT) | -.11/-.03 | -.18/-.34 | -.08/-.32 |
| Medial lemniscus R (RML)/L (LML) | -.15/-.02 | -.32/-.27 | -.17/-.26 |
| Pontine crossing tract (PCT) | **.59** | .47 | -.03 |
| Fornix (cres) R (RF)/L (LF) | .14/.17 | .02/.20 | -.14/.00 |
| Cingulum (hippocampus) L (LCH) | .07 | -.20 | -.29 |

L = left; R = right.

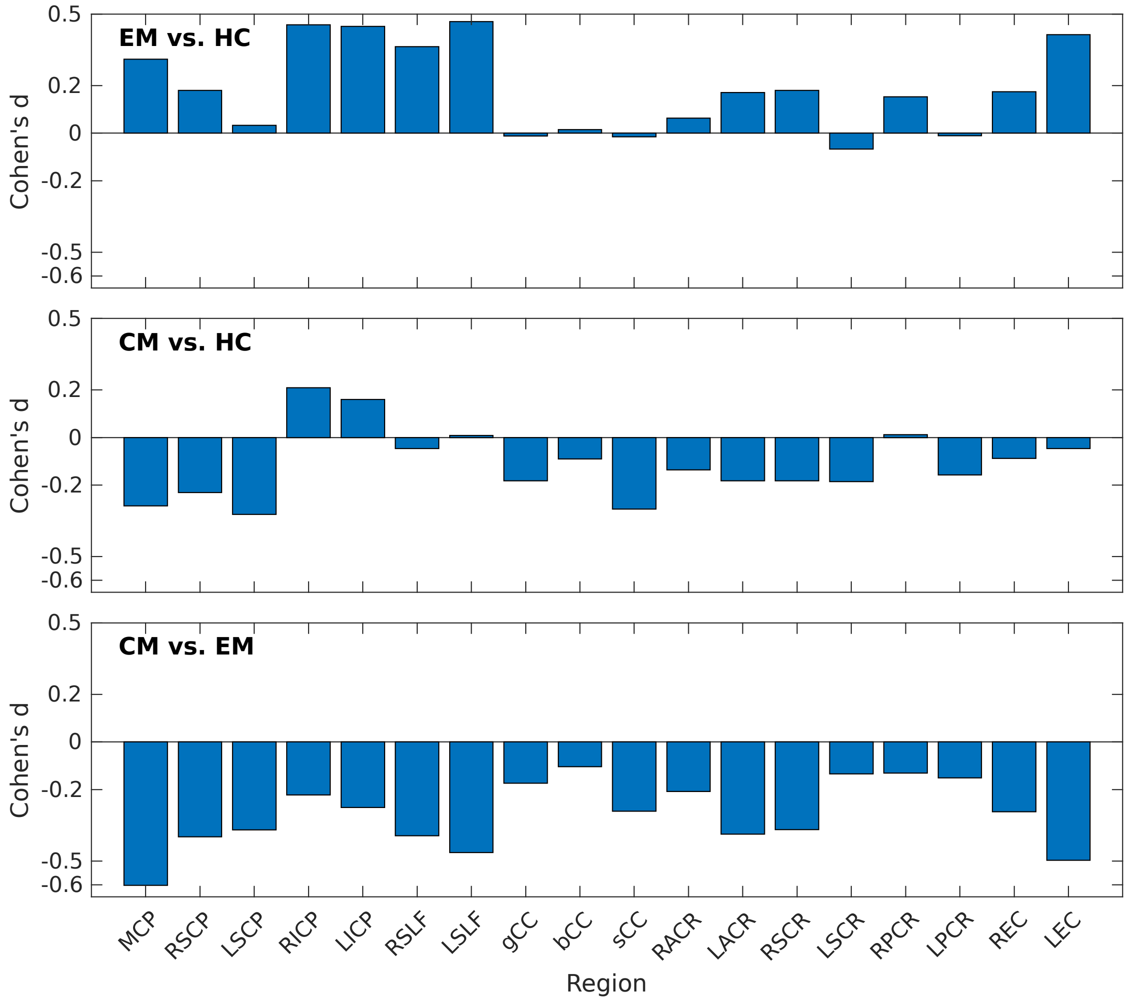


**Supplementary Figure 4 Cohen's AD bar plots of regions with FWE-corrected differences between EM and CM (part 1)**. Values for all possible pairwise comparisons are shown. Region abbreviations can be seen in Supplementary Table 7.

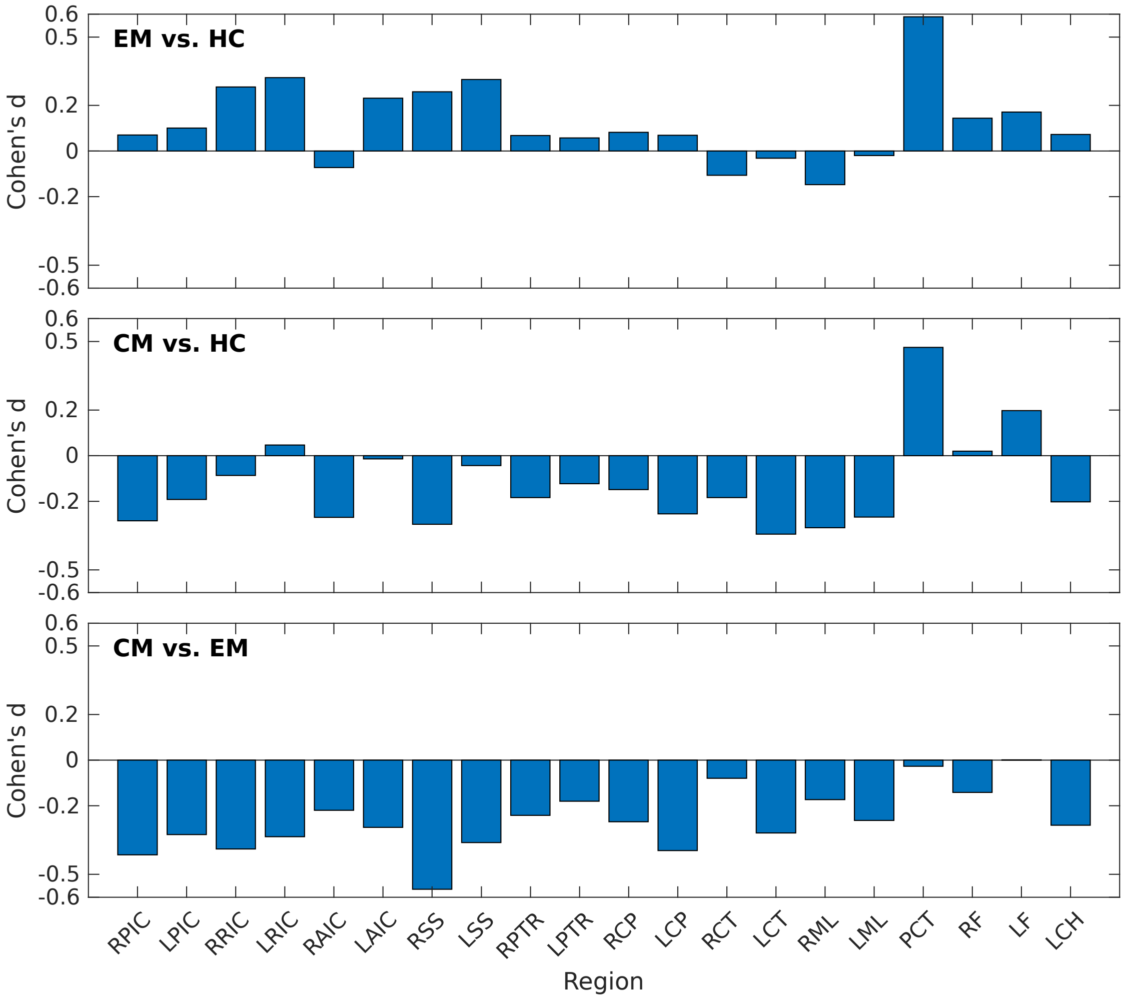


**Supplementary Figure 5 Cohen's AD bar plots of regions with FWE-corrected differences between EM and CM (part 2)**. Values for all possible pairwise comparisons are shown. Region abbreviations can be seen in Supplementary Table 7.

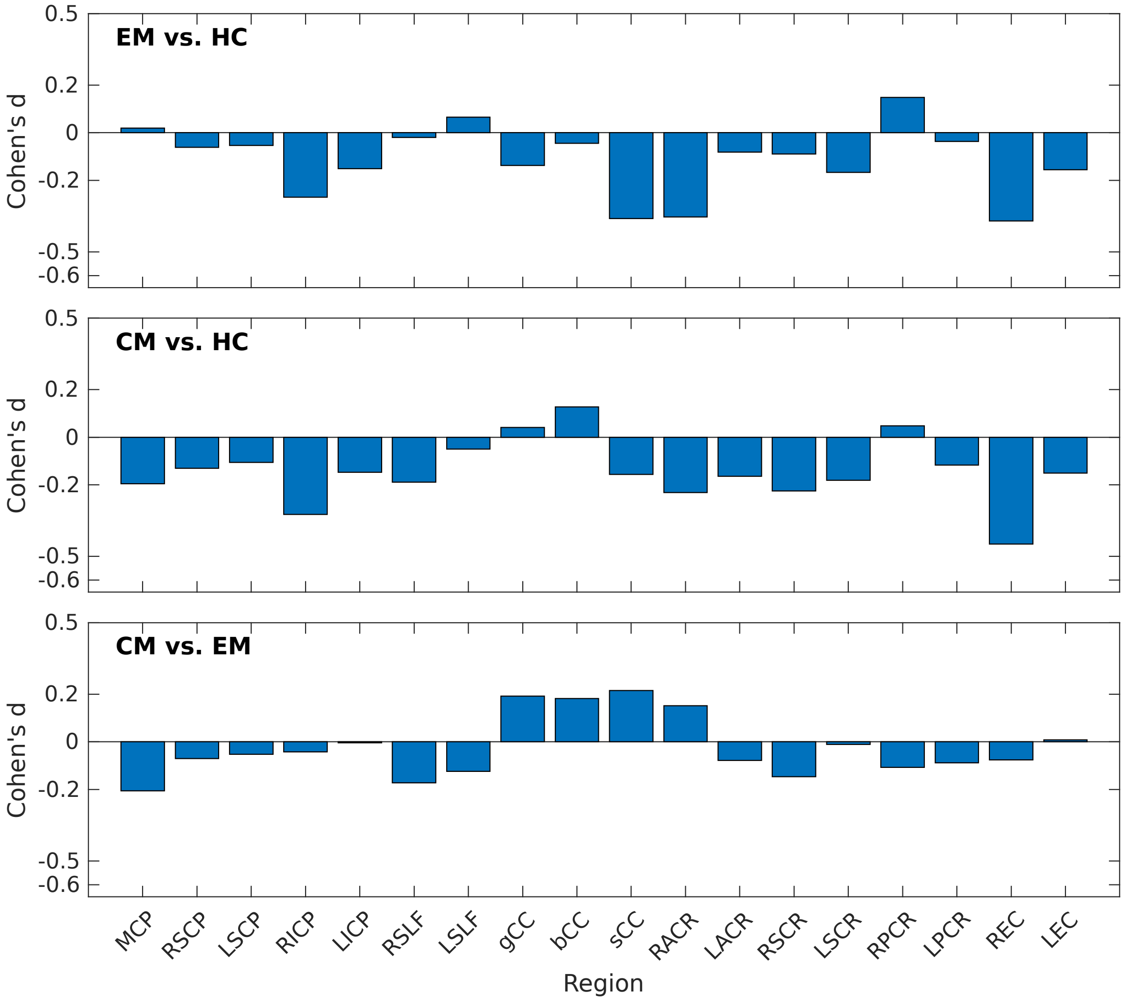


**Supplementary Figure 6 Cohen's d FA bar plots (part 1)**. Values for all possible pairwise comparisons where significant differences in AD between EM and CM patients were found are shown. Region abbreviations can be seen in Supplementary Table 7.

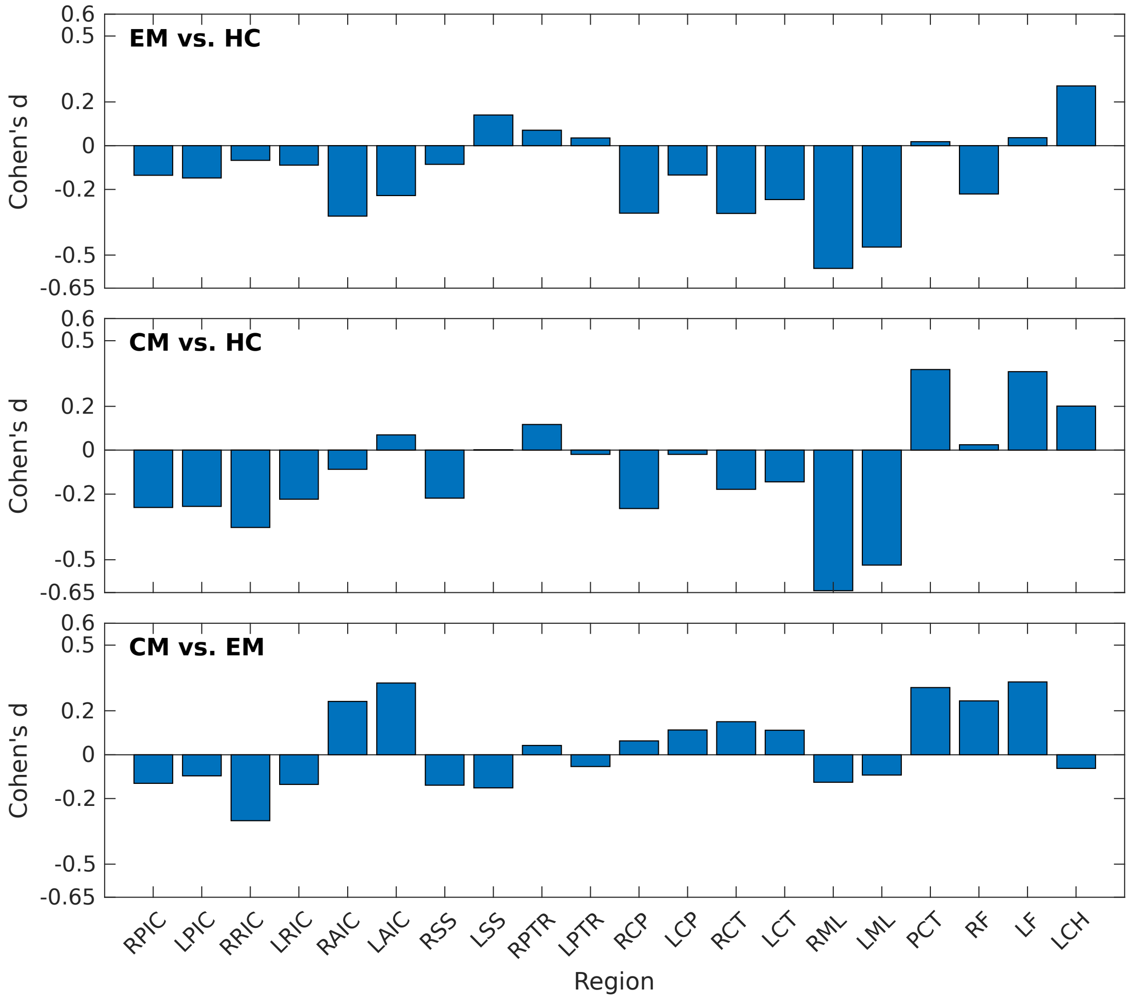


**Supplementary Figure 7 Cohen’s d FA bar plots (part 2)**. Values for all possible pairwise comparisons where significant differences in AD between EM and CM patients were found are shown. Region abbreviations can be seen in Supplementary Table 7.

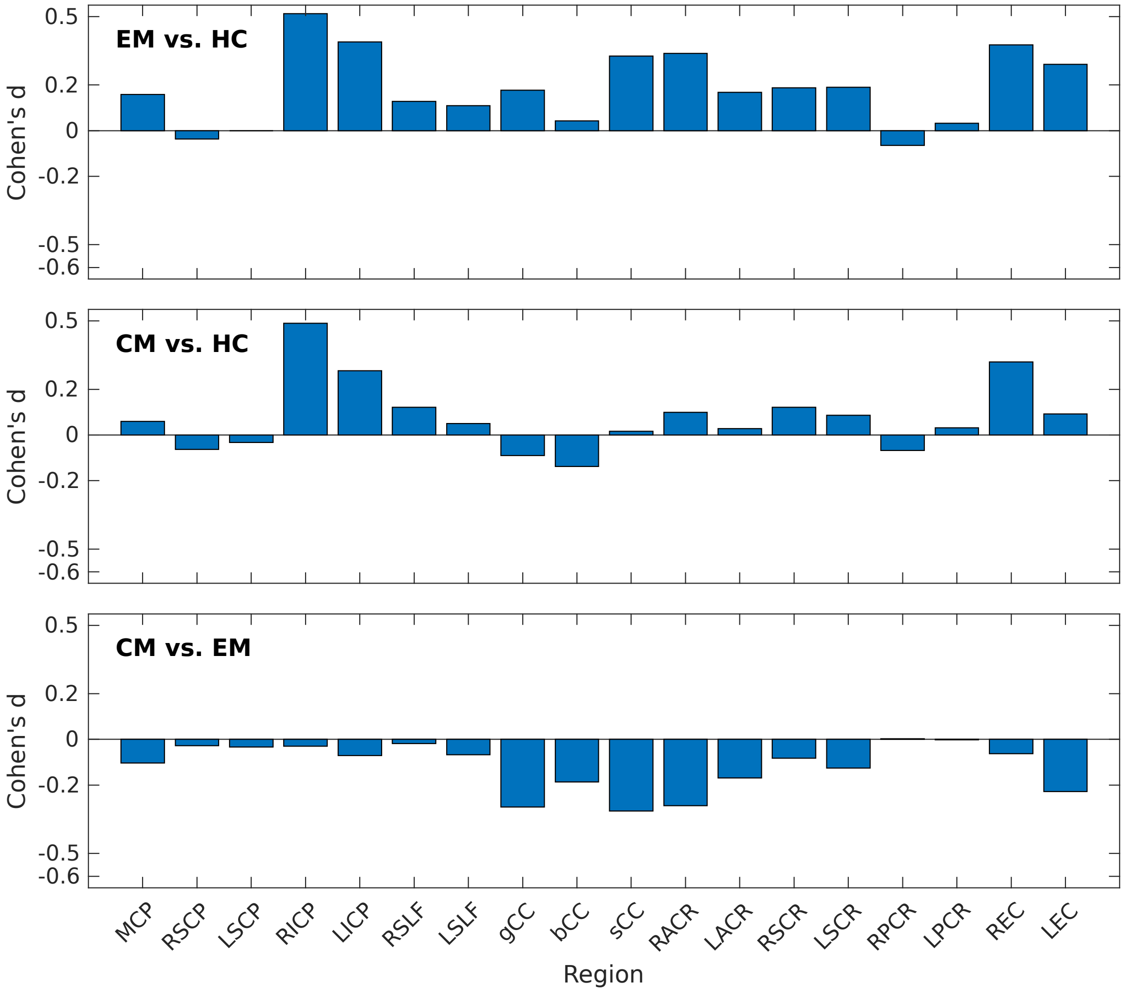


**Supplementary Figure 8 Cohen's d RD bar plots (part 1)**. Values for all possible pairwise comparisons where significant differences in AD between EM and CM patients were found are shown. Region abbreviations can be seen in Supplementary Table 7.

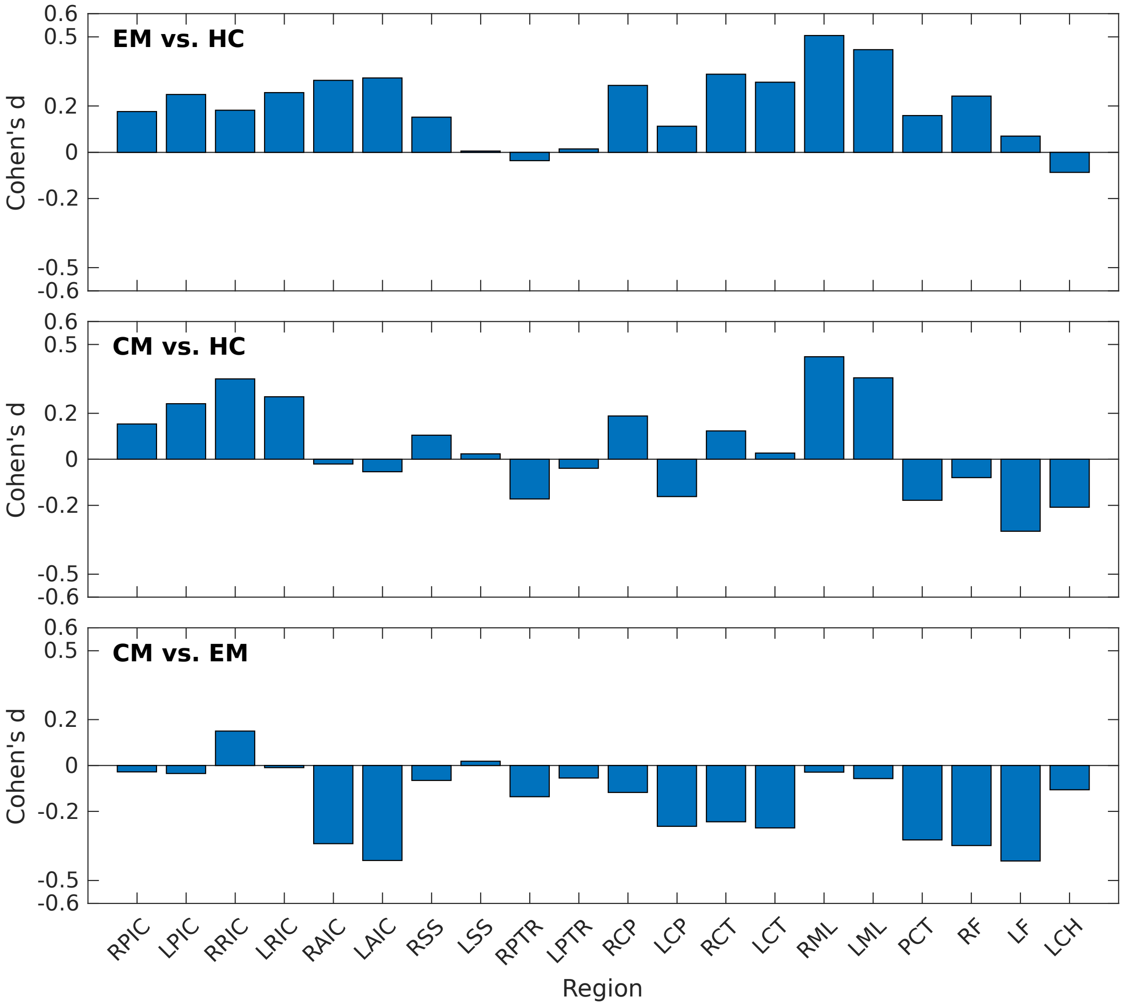


**Supplementary Figure 9 Cohen's d RD bar plots (part 2)**. Values for all possible pairwise comparisons where significant differences in AD between EM and CM patients were found are shown. Region abbreviations can be seen in Supplementary Table 7.

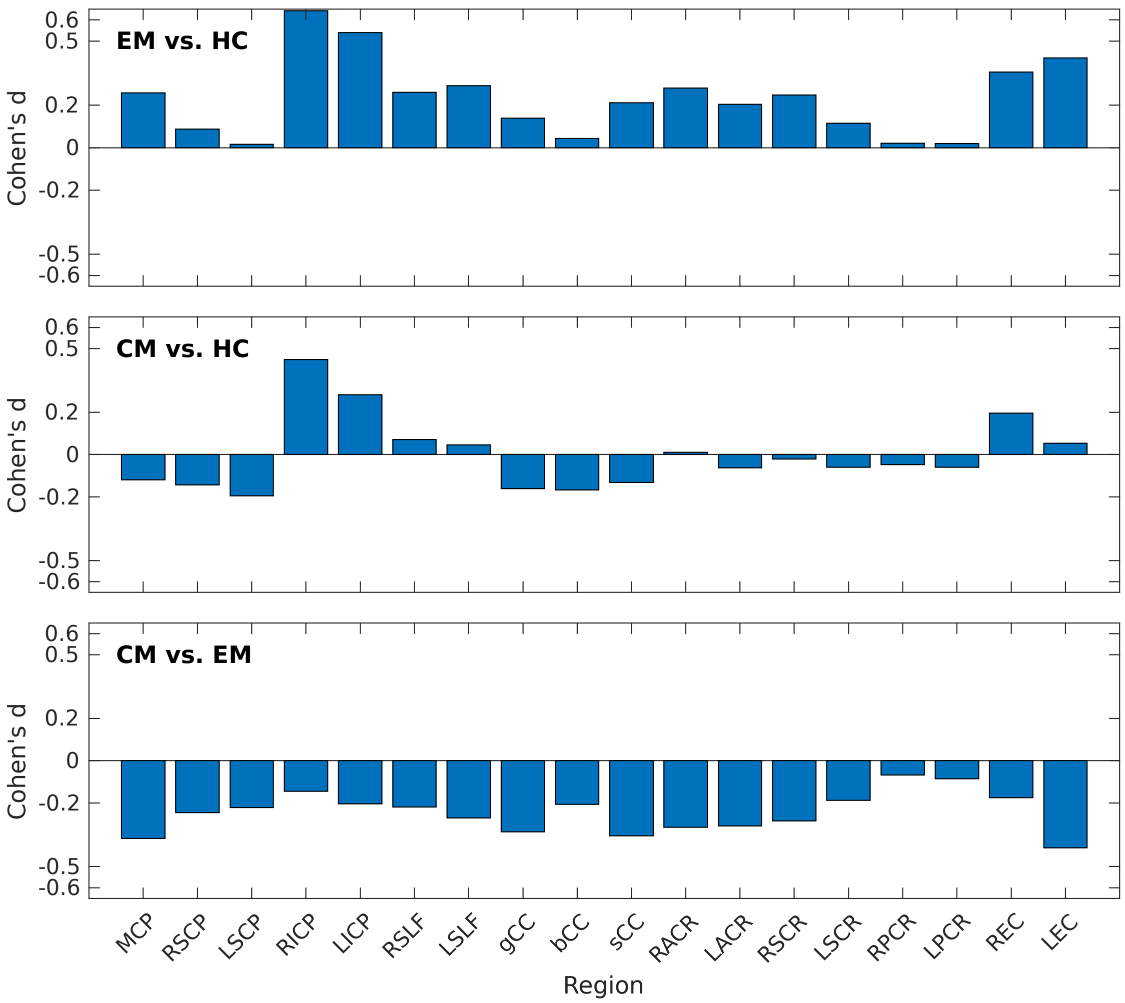


**Supplementary Figure 10 Cohen's d MD bar plots (part 1)**. Values for all possible pairwise comparisons where significant differences in AD between EM and CM patients were found are shown. Region abbreviations can be seen in Supplementary Table 7.

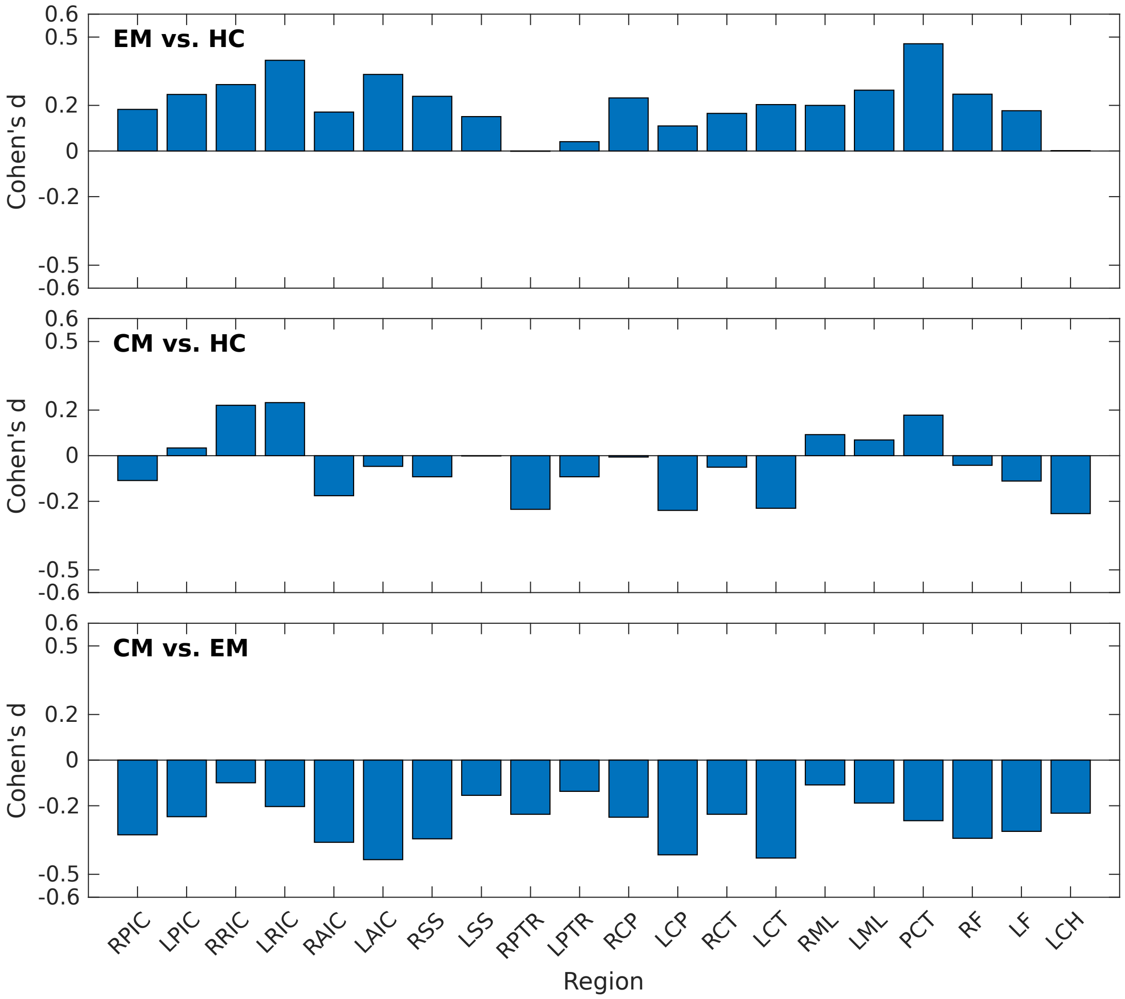


**Supplementary Figure 11 Cohen's d MD bar plots (part 2)**. Values for all possible pairwise comparisons where significant differences in AD between EM and CM patients were found are shown. Region abbreviations can be seen in Supplementary Table 7.
